# Medical Evidence for Mrs. Cumming

**Published:** 1852-04-01

**Authors:** 


### MEDICAL EVIDENCE FOR MRS. CUMMING.

*Robert Barnes,* M.D., examined by Mr. Serjeant WILKINS.—Q. I believe, sir, that you reside at No. 63, Gloucester Terrace, Hyde Park. A. I do. — Q. I think that for some years you have lectured on forensic medicine? A. Yes, I have. — Q. How long have you been in the profession? A. I have been studying it and in practise nearly twenty years—eight years in practice, and twelve in study. — Q. When did you first see Mrs. Cumming? A. At the time of the commission in 1846, at York House. — Q. I believe that at that time you visited her at the request of Mr. Haynes? A. I did. — Q. For what purpose did you visit her? A. I was requested by Mr. Haynes to visit Mrs. Cumming, I believe with this intention. He told me—

Sir FREDERICK THESIGER.—Never mind with what intention you visited her.

The Witness.—But I wish to explain the precise reason why I went.

Sir FREDERICK THESIGER.—I object to your stating the reason—it is quite immaterial what your reason was.

Mr. Serjeant WILKINS.—I submit that it was of the utmost materiality.

Sir FREDERICK THESIGER.—I must take my objection in form. His intention is a matter which must rest in his own mind. What passed with Mrs. Cumming is another thing.

The COMMISSIONER.—What his intention was it cannot be important to the jury to know.

Mr. Serjeant WILKINS.—I submit to you, sir, that it is most important.

The Witness.—I was requested—

Sir FREDERICK THESIGER.—You heard the commissioner decide that you were not to state with what intention you went.

Mr. Serjeant WILKINS.—I must not take anything *ex cathedrâ* that comes from my opponent. I submit to you, sir, in order to ascertain the accuracy and the amount of attention which this gentleman exhibited upon the occasion, it is of the greatest importance to know with what direct intention he went to that place.

The COMMISSIONER.—It is not necessary, at all events, in the present stage of his examination.

Mr. Serjeant WILKINS.—Very well, sir. — Q. At what time in the day did you visit her? A. I think on an afternoon. I can't be quite sure of the time of day. — Q. How long did the interview last? A. I first saw her for two hours. — Q. Can you remember the day, or not? A. I could, by referring to a note. — Q. How long did that interview last? A. Two hours; not less, I should think. — Q. First of all, let me ask this, what was the result of the opinion you formed after those two hours as to the state of her mind? A. The opinion I formed on my first interview was a cautious and guarded one. I did not wish to commit myself to a positive opinion. — Q. How many interviews had you with her? A. Three, certainly, before I became confident in my opinion. — Q. At the end of those three interviews, what opinion did you form as to the state of her mind? A. My decision was, that I could find no evidence of insanity; I was not prepared to say that she was not of unsound mind, but I could find no evidence to satisfy my mind that she was. — Q. Did you observe her deportment? A. I did, very attentively, and I could perceive no difference in her deportment from what I have ordinarily observed in other people in society. — Q. I do not know whether you were present at any time when the jury went to see her? A. I was present during the time of the commission. — Q. Were you present lately when any of the jury went to see her at all? A. No; I was not. — Q. Did you enter into any conversation with her? A. I did, at considerable length. I conversed with her on all occasions on the subject of the alleged evidences of insanity which had been advanced, and as to her past history, and her health especially, in order to see if anything had occurred in her past health which could have left any disposition to insanity, and I could discover none. — Q. Did you think all these inquiries essential? A. Yes.—Q. Did she talk rationally? A. Perfectly so. — Q. And calmly? A. Calmly, and with remarkable coherence and command over the flow of her ideas. — Q. Did she complain of the place? A. She complained very much of the associates she was obliged to mix with in the asylum. — Q. Did she complain of its being dull and gloomy? A. Very much so; she felt very much her being secluded as she was. She was accustomed to society, and it seemed to prey a good deal upon her mind. — Q. Did she complain of her friends being denied access to her? A. She did. She particularly mentioned that. — Q. Did she say anything about her anxiety? A. Yes; she said it was such that she had hardly had any sleep, or tasted any food since the commission had been adjourned three or four days before, and she was in great anxiety as to the result at that time. — Q. Were you present at any time when Mr. Haynes had a conference with her with reference to her property? A. I was. — Q. Did he at that time consult her as to the measures necessary for her defence for her case? A. He did. During the time I watched her attentively, in order to see how she behaved herself, and to see whether she understood clearly the questions which he put to her, and her position generally. — Q. Were papers and documents read to her? A. Several documents were put before her. I stopped her, and asked if she understood their contents and their nature, and she read them over herself attentively, and understood them fully, and assented to their propriety. — Q. You have attended her, and been acquainted with her occasionally from that time down to the present? A. I have, with a break perhaps of more than a year before I saw her in August last. — Q. Had you interviews with her on the 3rd and 6th of September, 1846? A. I had on those occasions. — Q. Did you inquire of her whether insanity had made its appearance in any other part of her family? A. I did. I traced every member of her family, and could get no evidence of anything of the kind; nothing that she could inform me of. — Q. When you were making inquiries as to whether there had been any symptoms in her family before that, did she say anything about her daughters? A. She made a remark which struck me. After going through all the other members of her family, and when speaking of her daughters, she said they were not insane, but that "the imputation of insanity might be as well transferred to them as be imputed to her." — Q. After she came out of the asylum, did she give you any particulars of excursions that she had made? A. She did. She said that she had been out from the asylum for a drive on two or three occasions. She seemed to be annoyed at the precautions by which she was surrounded when she was out. She said that one person would go out of the door first and see that she did not escape, and she seemed to feel that sort of annoyance very much. — Q. Did she say anything about being deterred from going in the front garden? A. Yes; she said she had been forbidden to go into the front garden. — Q. From the interviews which you have had with her up to this time do you believe her to be of sound or unsound mind? A. I may speak with all the confidence which one person can have when speaking of another after an intimate acquaintance, and I can speak to her soundness of mind on the same principles that I would speak of the soundness of mind of any person I might meet in the ordinary intercourse of society, my acquaintance with her extending now over a period of five years. — Q. Did she say anything to you as to her house being ill furnished? A. In 1846 she did. That was one of the grounds alleged, and I questioned her about it. She said her furniture was not safe where her husband was, and that she did not feel disposed to lay out any money in cutting up carpets in a place she did not mean to stay in. — Q. I believe that another topic urged against her was her aversion to her family. Did you probe her afterwards upon that point? A. I did. She principally referred on that occasion to the marriage of Mrs. Hooper, which seemed to rankle most in her mind, and she expressed at the same time a personal dislike to Mr. Ince; but with regard to her two daughters especially, my opinion was, that she did not entertain any decided animosity against them as her daughters individually, but it was chiefly on account of the dislike which she had taken to their husbands. — Q. Whom did she describe as the principal instigator of the proceedings which had been taken against her? A. She always referred to Mr. Ince. — Q. Did you speak to her about her property—about any neglect of her property? A. I did.—

Q. What was it that you said to her? A. She said that at that time that she had never had the means of repairing her property; that she had always been kept so close by the necessary expenses attending her husband's debts; and that she had taken some steps to have the repairs made when she was seized and carried away. — Q. Did she say that she was short of money? A. Yes, that she was always kept short by difficulties of one kind and another. — Q. Did she complain of Mr. Dangerfield? A. She told me that Mr. Dangerfield had deceived her, I think. — Q. Did she say whether she could get any account from him or not? A. Yes, that she could not get an account from him, or else that she could not get a satisfactory one. — Q. Did she say an account, or a satisfactory account? A. I cannot say; it was some dissatisfaction of that kind. — Q. Did she say anything to you about her husband's gallantries? A. Yes; she said that about fifteen years before that time (that was in 1846) her husband had an illegitimate child, and I questioned her as to his more recent gallantries, and she said that she did not impute to him connexion with the nurses in the ordinary sense of the word, but there were certain indelicacies which he was addicted to with them which she did not like to specify. — Q. Did you say anything to her about violent language which had been attributed to her? A. Yes; she told me she had always been rather of a hasty temper, and that when she was angry, she generally made people aware of it: that is what I heard from her. — Q. Did you say anything to her about her alleged habits of intoxication? A. With regard to that, she denied the imputation of being addicted to intoxication, and said that if it were true that she were addicted to intoxication the effects would be seen in her countenance, and she said it was very likely she would not confess it to me if she were. — Q. Did she complain of any servants? A. She complained of her servants—of having had many bad servants. — Q. Did you find that, although she seemed to brood over her wrongs, she had not any difficulty at all in detaching herself from those topics? A. None at all. She would turn to any subject which I mentioned to her with perfect facility. There seemed to be no engrossing attachment to any one idea or series of ideas. — Q. Had you an opportunity, at the inquisition on the 7th, and 8th, and 9th of September, of watching her demeanour during the sittings of that commission? A. I had. — Q. Did you hear her questioned upon different topics connected with herself and her property? A. I did. I heard the Commissioner's examination. — Q. What do you say of the answers which she gave to those inquiries? A. I found that her answers almost invariably agreed closely with what she had previously told me, and that they evinced the powers of memory, perception and attention to what was going on. — Q. Did you take notes of the evidence as it proceeded? A. No, not of that particular examination, but generally I did. — Q. At the time you have seen Mrs. Cumming at your different interviews, was there anything to intimate to you that she was a lady of dirty habits? A. Certainly not; I always assumed the contrary as far as I had opportunity of observing. — Q. Do you remember an interview she had with you on the 15th of September at York House, with Mr. Haynes, in your presence? A. I cannot be quite sure of the date. — Q. Do you remember her complaining of her health? A. Yes, I do. — Q. To what did you attribute that? A. To her confinement. She was then evidently suffering at that time from confinement and anxiety. Her health was beginning to shake the last time I saw her there. — Q. From the time of Mrs. Cumming's release from York House did you attend her as her medical man? A. I attended her constantly for the remainder of the year 1846, and throughout 1847, and I think I attended her a few times in 1848. — Q. Did you attend her at Mrs. Hutchinson's? A. I did; also at Camberwell-road, and at her present residence, St. John's Wood. — Q. Have you visited her lately in the present year? A. I saw her on the 25th of August last, at Worthing, and I have seen her four or five times since November last, since these proceedings. — Q. Have you a memorandum of a conversation which took place between you and her on the 25th of January, 1847? A. I have considered its purport pretty well. I have it not by me, but a conversation which seemed to indicate the state of her mind, and I took a note of it at the time. — Q. Do you remember the effect of it? A. She was afraid then, I think, of some proceedings or other, and she thought she had seen Mr. Hooper, I think, the night before, but she expressly stated afterwards she was not sure she had seen Mr. Hooper. — Q. Was that in consequence of anything you had said to her about keeping her gate locked? A. That gave rise to it. I was some time in getting in, and I asked her why the gate was locked, and why she was so careful, and she said she thought she had seen Mr. Hooper about there, but she was not sure of it. She said she was aware that her mind had become unusually suspicious in consequence of what had occurred to her, and she might fancy that she had seen what she was afraid of seeing. She seemed to me to have a watchful care over her mind, and a power of analysis which seemed to me to be a strong indication of a sane mind. — Q. Has Mrs. Cumming conversed with you on the subject of her property? A. Occasionally. — Q. Has she talked to you about her property in Wales, and as to the difficulty of getting her rents? A. She has mentioned it from time to time. — Q. Did she say anything to you about her children's claim to her affections? A. She seemed to think that they had forfeited all claim to her natural affection. — Q. Did she say anything to you about her grandchildren? A. I think that was the only occasion on which I suggested anything to her with regard to her property. I said that her grandchildren were innocent, that they, at all events, could not have given her any cause of offence, and I suggested, in as delicate a way as I could, that she need not extend her animosity to them, that she might make some provision for them. — Q. What did she say to that? A. Her answer was very short—that the grandchildren must take their lot with the children; that the object of her children would be partly accomplished if they could effect such a settlement upon the grandchildren, and that she would not be coerced into any proceeding of the kind.

The COMMISSIONER.—Do you say that their object would be answered, or partly answered? A. Answered, or partly answered.

Mr. Serjeant WILKINS.—Did she say anything about visiting the sins of the parents upon the children? A. Yes; she made use of an expression of that kind, that the sins of the fathers should be visited on the children.

A JURYMAN.—That they were? A. That they were, or should be; I will not pledge myself to the exact words. That was the expression used.

Mr. Serjeant WILKINS.—I believe that through the month of September, 1847, you were attending her for an attack of bronchitis? A. I was. — Q. Do you remember on the 5th of October, the following month, finding her in bed? A. I do. — Q. On that morning did she say anything to you about poisoning fowls? A. Either on that morning or on one or two mornings previously. I cannot remember the exact day on which the fowl was found dead. — Q. What did she say —did she say anything about the milk? A. On the 5th of October there was some milk in the room, which they had offered to the cats, but which they had refused to drink.

Sir F. THESIGER.—She said so? A. She told me this herself.

Mr. Serjeant WILKINS.—And she said that the cats had refused to drink it? A. Yes, that they had refused to drink it. That was after the fowl had been found dead. — Q. The fowl had been found dead a morning or two before? A. A morning or two before. — Q. By her instructions did the servant give you a packet of crystalline substance, which was said to have been found in the fowl house? A. She did; and I inferred that it was found there because it was stained with fowl's dung. — Q. The paper was? A. The paper was which contained it. — Q. Was some milk also shown to you, in a saucer? A. Yes, it was. — Q. Did you pour that in a clean bottle? A. I put it in a clean bottle, and took it home with me, at Mrs. Cumming's request. — Q. What did you find the crystalline substance to be which was given you in the paper? A. That was acetate of lead. — Q. And what was that which was found in the milk? A. It contained a considerable quantity of Epsom salts. — Q. What does acetate of lead look like in its crystalline form? A. There are three or four crystalline substances which are very much alike—it is like Epsom salts. — Q. Would the acetate of lead given to the fowls be in appearance like the salts found in the milk? A. In its natural state it would, and it would also be like oxalic acid—they have all a general appearance very similar. — Q. At that time, did Mrs. Cumming appear to you to be very much excited or not? A. She certainly was very much agitated, and very much annoyed at the occurrence. — Q. When did you tell her it was acetate of lead? A. A morning or two afterwards; when I had examined it. — Q. What did she say about it? A. She, at first, was inclined to think that her family had been the means of having it placed there to kill the fowls, for the purpose of annoying her. — Q. Did you afterwards ascertain how it was the Epsom salts came to be found in the milk? A. No, I did not; not that I remember—I merely remember the fact of examining it. — Q. I believe that, from the beginning of 1848, Mrs. Cumming has been attended either by Dr. Hale or Dr. Caldwell? A. I believe so. — Q. Did you also visit her occasionally in your professional capacity down to the end of June in that year? A. Yes, in 1848, I did occasionally, but very seldom. — Q. Have you occasionally, during the years 1849 and 1850, visited her? A. I have when I have been in the neighbourhood, I have called upon her, and she has sometimes called upon me. — Q. Did you observe anything in her conduct and conversation different from that of a sane person? A. Not at all. — Q. Did you notice whether she maintained perfect control over her own household, and over her own affairs? A. I did; and I am perfectly satisfied that she did, from my observation. Everybody in the house was attentive to her orders, and solicitous about her. — Q. Did any one, as far as you could discover, treat her as a person incompetent to manage her own affairs? A. Not that I ever observed. — Q. Was she somewhat of an imperious disposition? A. She certainly was. — Q. Did she exact much attention from her servants? A. A very great deal; and I have no doubt it was often annoying to them. — Q. Did it appear to you that she was sometimes more authoritative than there was any occasion for? A. Yes, I certainly observed that. — Q. From the middle of 1850, to August 1851, you did not see Mrs. Cumming at all, I suppose? A. I think not; for more than a year I lost sight of her altogether. — Q. On the 25th of August you visited her at Worthing? A. Yes. — Q. Was that at her request? A. I understood it was at her request. — Q. When you got there, did she say anything about her family? A. She said she was at Worthing in order to escape from their pursuit—that she was then under the feigned name of Mrs. Cleveland, the better to avoid detection. — Q. Did you find Dr. Caldwell there? A. Dr. Caldwell was there when I arrived. — Q. I believe you waited until his interview was over? A. I did. — Q. Did you then go in her bed-room? A. I did. — Q. Was she dressed? A. She was dressed neatly, as usual. — Q. How was her health then? A. It was very good, comparatively speaking; I had seldom seen her better, in my judgment. — Q. Did you then enter into a conversation with her upon the proceedings that were taking place against her? A. I did; she referred to it herself. — Q. Did she talk clearly concerning them? A. Quite. — Q. Did she tell you of any measure she had taken to escape from her relations? A. She did; she told me that she was alarmed at some irruptions into her house. I cannot precisely fix what the irruptions were that she alluded to, and said she was very uneasy at staying in the house where she was, and that she had got up early one morning, I think at seven o'clock, and went to ask Mr. Haynes' assistance to get away from the place. — Q. Did she relate to you a visit from Mrs. Ince to her residence in the Edgware Road? A. She did; I can't say that it was in the Edgware Road, but she related an interview with Mrs. Ince. — Q. Would you have the goodness to describe it? A. She said that while some proceedings were being taken, Mrs. Ince rushed suddenly into her room, without any announcement or preparation; that she was alarmed at her entrance, and that Mrs. Ince threw her arms round her neck; that her first impulse was that Mrs. Ince intended to do her violence, she was so little prepared for any act of apparent affection; but she told me plainly that she afterwards dismissed that idea, and attributed it to the anxiety and alarm which she was under from the conduct of her children. — Q. That was at the same interview? A. At the same interview; she dismissed the idea, knowing her mind to be in such a state that she might be attributing motives which perhaps were not just. — Q. You say she got up one morning at seven o'clock to send for Mr. Haynes? A. To go to Mr. Haynes, if I understood her rightly. I am not sure how that was. — Q. Did she say why she had come to Worthing? A. To escape from her family. — Q. Did she say anything about the length of time she had been kept in a state of alarm? A. She seemed seldom to have been altogether free since the time of the first commission. — Q. Did she say whether she had requested any person to stay with her? A. Yes, she did. — Q. Who did she say she had requested to stay with her? A. Mr. Jones, who was going there by the name of James. — Q. Did she say why she wished him to stay with her? A. She said she felt desirous to have some one by her who would be watchful, and in a condition to protect her. — Q. Did you say anything to her about the protection of the law? A. I explained to her, generally, that the law would protect her, and that she would have nothing now to fear—that any steps that could be taken would be taken under legal authority. She said I

might have told her that five years before—at the time of the former commission. She said they had taken measures against her then, and would probably do it again. — Q. Did she say anything about her courage? A. When I took leave of her, I said she might have occasion for further courage to undergo any further trial. She said she "hoped she should be equal to it, but constant dropping would wear away a stone." — Q. Did she say anything about her determination to resist? A. Yes, she said she would resist to the last. — Q. Some time after this, had you an interview with her in the presence of Mr. Hutchinson and Mr. Jones? A. Yes. — Q. On that occasion did she appear to you to be mistress? A. Perfectly. — Q. And to have control over all the persons in the house? A. Perfectly so. I had the fullest opportunity of seeing that, and was perfectly sure of it. — Q. On the 2nd of September, I believe you attended the Board of the Commissioners? A. I did. — Q. Where was that? A. At Spring-gardens. — Q. I believe you made a statement to them? A. I made a statement to them of the interview I have just mentioned at Worthing.— Q. On the 27th of November, did you visit Mrs. Cumming at the house at St. John's-wood, after her release from the Brixton asylum? A. Yes, I did. — Q. Was she sitting by the fire in the bed-room? A. She was on that occasion. — Q. You had seen her, you know, before she was taken? A. I had, on the 25th of August. — Q. And you saw her again on the 27th of November? A. Yes. — Q. Was she then altered or not? A. She was very much altered, indeed, for the worse; her health was shattered in a great degree; and not only her health but her mind was evidently also affected to a certain extent. — Q. Do you mean that her memory failed her? A. Her memory—that is what I chiefly observed; for a time she was a little confused, and also a little disturbed. — Q. Did she possess animation and readiness on that occasion in conversation? A. She was glad to see me; towards the end of the time she seemed to recover a little; as she regained confidence, and had got a little better, she conversed with much more fluency and accuracy than she did at the beginning of the interview, and I could see a difference even then. — Q. Did she recount to you all that had passed to her since you had seen her before? A. She recounted the fact of her having been taken to the asylum, and generally what had taken place. — Q. Although you discovered this alteration in her memory, did that impairment of memory amount to anything like insanity? A. Most decidedly not; I consider it, to a great extent, the result of her recent confinement, her severe illness, and the mental shock she must have undergone on that occasion. I considered I was fully borne out, in this conclusion, on seeing her subsequently, on the 30th of November, and once or twice in December, when I found her recovering still more, and when her memory, which had been impaired on the 27th, was coming round very much as her health improved. — Q. Did you see her on the 17th of December? A. Yes. — Q. Did you find an improvement then? A. Yes. — Q. Did you visit her on the 9th of December, 1851? A. Yes, I did. — Q. Did you still find a progressive improvement in her health? A. Yes; she was still improving. — Q. On the day that you called, did you ascertain that Dr. Diamond and Dr. Davey had seen her in the morning? A. She told me so. — Q. Although her memory is improving, still is it defective with regard to recent events? A. I should say it is. — Q. Do you think that at all material? A. It is what every one must anticipate—it is quite natural. — Q. In proportion as her health has improved, and personal comforts have increased around her, have her anxieties since that diminished? A. They have. — Q. And has her mental vigour returned? A. Certainly. — Q. Is it your opinion that, supposing her mind was released from the anxiety attendant on an inquiry like this, she would recover her usual equanimity and intellect? A. I think she would entirely, except so far as age may tell. — Q. We are told that your name is mentioned in her will—when did you first ascertain that? A. In August, 1851. — Q. Had you never heard it hinted at before then? A. Never; I was quite ignorant of any will that she had made, or the contents of it. — Q. I believe you declined being executor when you did hear of it? A. I did. — Q. Have you a list of the different times that you visited Mrs. Cumming? A. I have—producing a paper—(the witness recited the dates of his visits to Mrs. Cumming from September 1846 to June 1848, amounting, altogether, to about one hundred visits). — Q. Then you saw her at Worthing? A. August 25th, 1851; November 27th and 30th; December 17th and 29th.

Mr. Serjeant Wilkins.—When was the last time you visited Mrs. Cumming? A. I saw her, for a few minutes, last night. — Q. Did you converse with her then?

A. Shortly. — Q. Is your opinion unchanged: are you still of opinion that she is of sound mind? A. Quite. I am quite of that opinion. — Q. On all occasions when you have visited Mrs. Cumming, whether she has been in her room, or in any room, have you found her neat, clean, and orderly in her person and in her house? A. Always.

Cross-examined by Sir F. THESIGER.—When did you become a doctor of medicine? A. I became a doctor of medicine in 1848. I was a bachelor of medicine in 1843. — Q. Have you had much experience in cases of insanity, or has your practice been more general? A. I have had considerable opportunities of observing insane persons. — Q. From what time should you say? A. During three years that I was a pupil I was constantly in the habit of seeing a pauper asylum containing forty patients; and I was subsequently a student at the Salpetrière, in Paris, for a whole year. — Q. And during your pupilage you had opportunities of seeing cases of this kind? A. Constantly. — Q. Since you have been in practice for yourself, have you had experience in cases of insanity? A. Not especially; it is since I have taken my diploma that I have had the opportunity which I have spoken of in Paris. — Q. But it is not your particular calling? A. It is not my particular calling. — Q. You probably will be able to give us your definition of a delusion; what do you consider a delusion? A. Persons are suffering under delusions when they believe in things that do not exist, and draw wrong inferences which are not justified by the premises. But insane delusion is also associated with a diseased mind, depending on a diseased state of the brain. — Q. That is the cause; I am asking you to give me a definition of a delusion; is the definition of a delusion believing that which is not true, and drawing wrong inferences from it? A. That would not constitute an insane delusion; certainly not. — Q. Is it not rather believing things which do not exist, and drawing right conclusions from them, that is, drawing conclusions from them as though they did exist? A. Not necessarily. I should not take that as an indication of insanity, not even the existence of a delusion absolutely. — Q. You do not consider a person labouring under a delusion is in an unsound state of mind? A. Not necessarily; there must be some evidence of a diseased judgment; there must be diseased operations of the mind as well. — Q. Do you mean, that when a person is labouring under a delusion, that is, under the belief of that which is not the fact, and which never existed, is not in a diseased state of mind? A. Not necessarily so. — Q. Is it your belief, that a person may labour under a delusion—under the most striking delusion imaginable—and yet may be all the while of a sound mind? A. I would instance the Mormonites, or any other general delusion; we are all under some delusion or other. — Q. Then it is your opinion that there is not such a thing as a sound mind? A. No, I have said nothing of the kind. — Q. What is your particular delusion? A. I say, a delusion in itself is no test of insanity. — Q. As we are all under a delusion, may I ask what is your delusion? A. I do not feel called upon to expose my own infirmities. — Q. But you have delusions? A. No doubt; I do not pretend to be wiser than others. — Q. What did you receive for going to Worthing? A. I have not received any fee for that; of course, I expect my expenses; but there is nothing else due. I have no clear expectations at all. — Q. I merely want to know whether you have been paid your fee, or not—you are to be paid for going to Worthing? A. I have not been paid for going to Worthing. — Q. You will have what is right, no doubt; how much are you to have, how much do you expect? A. I do not know that I can be fairly called upon to state what fee is usual for a physician going to Worthing.

Mr. Serjeant WILKINS.—Pray, do not object to it.

WITNESS.—I have no objection to state it; but it appears to me to be a question not relevant to the point. I will state, that whatever I expect will be the usual fee, and no more. — Q. I understood you to say that you were attending Mrs. Cumming in the year 1847, and that there were various occasions on which you did attend her, and saw her, and had conversations with her at those times; is it so? A. Those are the dates of my professional attendances. — Q. When you paid your professional visits, did you talk on other subjects with her? A. Yes, I very often talked on other subjects, of course. — Q. Attending her in the years 1847 and 1848, am I to understand that Mrs. Cumming ever spoke to you at all about her will? A. Never, except perhaps the occasion I have referred to when I talked of her grandchildren; but she never consulted in any way on the subject. — Q. She never asked you to be trustee, did she? A. No, she never asked me that. — Q. Or executor? A. No, she never asked me to be her executor. — Q. She never told you that you were a legatee under her will? A. Never. — Q. You say, that on the 5th of October, 1847, she told you about the milk; you say, you ascertained that that which was said to come out of the milk contained Epsom salts; and that in what was represented as having been found in the fowl-house there was acetate of lead? A. Yes. — Q. Who analyzed it with you? A. The acetate of lead being in a substance, the analysis is easy, requiring only about five minutes, and I did it myself. With reference to the milk, I was very much engaged at the time, and I thought the milk might change a little; I gave half of it to a Mr. Spencer, whom I had known at the College of Chemistry, where we had been students together, to analyze for me; and I analyzed the other half on another occasion. — Q. Did you tell Mrs. Cumming that the milk contained Epsom salts? A. I have very little doubt that I told her what I found. — Q. Did you tell her that the milk contained Epsom salts, and that the other matter found in the fowl-house contained acetate of lead? A. I have no doubt of it at all. — Q. Are you sure that it was on the 5th of October, 1847, that this happened? A. That is the date I have. — Q. You have made an affidavit upon this matter, and there is a copy of it (handing the same to the witness); look at it, and say whether you did not state that it was the 5th of October, 1846? A. If that is so, it must have been an error in copying, for in 1846 she was in Camberwell, and at Mr. Hutchinson's, and this was in the Queen's-road. — Q. You are sure it must have been in the Queen's-road? A. Yes. — Q. Did she refer to this circumstance about the milk afterwards? A. She did, once or twice afterwards. — Q. What did she say about the milk at that time? A. She said, that she thought it was done to annoy her, and to irritate her. — Q. Did she say, she thought it was done to poison her? A. No, she did not say that. — Q. Then she was quite satisfied at those subsequent times, at all events, that there was no poisonous substance in the milk? A. She might, and did, forget sometimes that the acetate of lead was a distinct substance, and that it was not that that was found in the milk. — Q. But at other times did she understand that there was no poisonous substance in the milk at all? A. I am not sure that she did understand thoroughly; it is some time ago, and I do not recollect the conversations accurately. — Q. Suppose she talked to you on the subject of the milk, and you found her under the impression that there had been poison in it, did you endeavour to remove the impression? A. I have no doubt I should have corrected the impression. — Q. I understand you to say, that in one of your interviews with Mrs. Cumming she spoke of her daughter rushing into the room in the Edgware-road, and embracing her; that she believed, at first, she meant to strangle her, but that her impression was removed when she came to consider, and that nothing of the kind could be intended? A. Yes. — Q. Her first impression was that there might have been such an intention? but you say that that impression was entirely removed from her mind? A. Yes; on subsequent consideration. — Q. Will you be good enough to tell me when it was she said so? A. That was on the 25th of August, 1851, when she was relating the whole circumstance to me—she related it in that way. — Q. Has she since that time ever spoken to you upon the subject of her daughter's visit to the Edgware-road? A. Yes, on a subsequent occasion she mentioned it again. — Q. What has she said to you upon the subject? A. She has given very much the same account—substantially the same account. — Q. Then she has never since that time stated to you that her daughter intended to strangle her? A. No; she never stated it to me in such a way as to lead me to suppose that she entertained that idea now. — Q. That is, then, she never stated to you anything to lead to the impression on your mind that she believed her daughter was going to strangle her? A. Not that she believed it at the present hour, or that she believed that, at the time I spoke to her. — Q. Do you believe that she ever entertained that impression? A. Only during the time of the occurrence. — Q. Then at that time when you saw her she entertained that notion? A. No. — Q. But she has invariably told you pretty much the same thing—that although she might at first have believed it, yet, on consideration, she thinks she must have been wrong—that it must have been her suspicious mind? A. That is the account she gave of it. — Q. Has that been invariably the tenor of her remarks? A. I do not remember any other account of it. — Q. Now, suppose she told you she was persuaded in her mind that her daughter had attempted to strangle her, and asserted it over and over again, would you have considered that a delusion, or not? A. I do not know that I should, if she were prevailed on by reasoning a second time afterwards, to dismiss the idea. — Q. Your opinion is, that if she confidently asserted to others, over and over again, that her daughter did intend to strangle her, that is not a delusion, and indicates no unsoundness of mind? A. It might be a delusion. — Q. But not an unsoundness of mind? A. Not necessarily an insane mind. — Q. Does it indicate an unsoundness of mind? A. What I mean by an insane mind, is a mind under the influence of disease—a disease of the brain. — Q. A disease of the mind is a metaphorical expression; what do you mean by a disease of the mind? is a mind, which is not in a sound and healthy state, diseased or not, in your estimation? A. A mind that is not in a sound condition may be supposed to be diseased. — Q. Do you consider a mind, labouring under a delusion, to be in a sound condition, or not? A. Under the ordinary acceptation of the words, a person may be in a sound mind, not diseased, and yet under a delusion. — Q. Then do I understand you to say, that you consider that although a person is labouring under a delusion, he may still be of a sound mind? A. Taking the general phrase I would certainly say that; but not an insane delusion—I would make that distinction. — Q. What do you mean by an insane delusion? A. A delusion occurring in an insane mind. — Q. You will distinguish between a delusion and an insane delusion; will you tell to the jury what distinction you make between the two? A. It is difficult to do it. — Q. Explain, if you please, the difference between a delusion and an insane delusion? A. It is difficult to do it; but what I understand by an insane delusion is, one influenced by some striking lesion of the ordinary processes of thinking. — Q. But there again you get figurative; what do you mean by lesion of the ordinary processes of thought? A. When you observe that a person shows some breach of the ordinary connexion between cause and effect, which is different from what ordinary persons exhibit; it is not a purely metaphysical question, but what we observe in the daily occurrences of life. — Q. I want you to give us the distinction which you have given, because that is one which I do not understand, and which my learned friend, Mr. Serjeant Wilkins, does. A. I think those forms of delusions are very rare where the general faculties of the mind are not obviously impaired in other matters as well. — Q. You consider, then, an insane delusion is when there is a lesion of the mind; is that your distinction? A. A lesion in the reasoning process. — Q. A lesion in the reasoning process, which is a break, or rupture, if I may venture to call it so, in the reasoning process? A. I will not adopt your expressions. — Q. Is it a wound in the reasoning process?

A Juryman.—Will the Doctor inform us what he means by lesion?

Witness.—It is something very different from what we observe in the reasoning powers of ordinary individuals, from which we get our opinion, perhaps a very general one, of soundness of mind. — Q. Lesion is a word which has a meaning, has it not? A. Yes. — Q. Yes; and it has a meaning in your mind; will you give us the meaning of the word lesion, as you use it? A. A distortion, or disease; something strikingly different from what is observed in ordinary individuals. — Q. Is lesion a distortion or disease? A. It is the result of a disease; you may take the word you use—a hurt if you like. — Q. A break, or hurt, or a wound, is it not? I know that a definition is a perilous thing. A. I do not consider that in forming my opinion of the sanity of a person I am bound to enter into definitions; I formed my opinion of Mrs. Cumming's sanity upon the same grounds and principles as those which I should apply in any other case. — Q. You are not here to form an opinion as to the sanity of Mrs. Cumming, but to give your opinion to the jury, in order that they may form their judgment upon it. A. I have formed it from having observed the operations of her mind, and they are in no respect different from those which I observe in other persons every day. — Q. Then do you mean to say that because you have found Mrs. Cumming rational on many subjects, although she may be labouring under various delusions, if there is no lesion she is perfectly sane? A. I do not think I am bound to take your hypothesis, for I do not think she does labour under those delusions. — Q. But you must take my hypothesis, as the question I put to you is one of science; I ask you, supposing it should turn out that Mrs. Cumming was labouring under several delusions, although she might be perfectly rational upon many other subjects, and most other subjects, is it your opinion that, notwithstanding those delusions, she is a person of a sound mind?

A. I should like to be certified, before I gave an affirmative opinion on that point, as to the nature of the particular delusions, and I could not answer the question generally. — Q. I will take the idea that her daughters are going to murder her, and the idea repeated over and over again—I will take that? A. I am not sure that I can admit that, if I disassociate it from any other evidence of a diseased mind; I do not see how I can admit it. — Q. How do you mean disassociate it from any other idea of a diseased mind? A. I can find nothing in her mode of thought different from that of sane persons; and I cannot see, because a person fears that another is going to murder her, that in itself is a sign of her insanity. — Q. Do you think it is no ground for it? A. That I can say nothing about; what may appear grounds to one person may not appear grounds to another. — Q. Then you consider that if there is any, the slightest occasion, upon which any suspicion may arise in the mind of a person, that would justify a belief that the person against whom the suspicion is entertained is capable of any act however atrocious against her? A. Not *necessarily*; it might, or might not, according to the tone and disposition of the person in whom that cause might operate; some persons entertain the most violent and outrageous conceptions from a cause which would not strike another as deserving of attention. — Q. You consider that it is a mere difference of mind, that where a person entertains these extraordinary suspicions, without anything but the slightest foundation, that is merely the particular character of the mind? A. It is not necessarily a proof of insanity—certainly not. — Q. Then I understand you to say, that where delusions prevail on particular subjects, that is not necessarily a proof of insanity? A. Certainly, you may take that as my opinion. — Q. Suppose the delusion should be as utterly groundless as possible—suppose it has nothing whatever to rest upon, what would be your opinion then? A. It might show some impairment of mind. — Q. It might show some impairment of mind? A. It is impossible to state these things generally. — Q. You qualify so much? A. I must qualify to a certain extent. — Q. Would it show unsoundness of mind? A. Every impairment indicates unsoundness of mind, but there are degrees of that. — Q. Give me my own word which I put to you, which is, unsoundness of mind; why shift the term. Would it in your judgment be considered unsoundness of mind? A. It might with that qualification. — Q. With what qualification? A. A certain degree of unsoundness of mind might not indicate insanity, that is, not necessarily. — Q. A certain degree of unsoundness of mind, what do you mean by that? A. There are different degrees and different stages of insanity, and different varieties of insanity. — Q. Do you mean that a mind can be sound and unsound at the same time? — A. What I have stated does not involve that absurdity. — Q. Is it your judgment that a mind may be sound and unsound at the same time; I want to know that? A. I think there is in every mind soundnesss and unsoundness. — Q. Is it your opinion that a mind may be sound and unsound at the same time? A. If you will take my answer in the words I give you, I will adhere to that answer; there is some soundness and some unsoundness in every man, inasmuch as no mind can be perfect. — Q. No doubt some parts of the mind may be sounder than others, but I am speaking of a mind which is diseased, and I ask you, whether it is your opinion that a mind has been sound and unsound at the same time? A. If by an unsound mind you mean a diseased mind, you beg the question. — Q. I am obliged to beg the question, and beg very hard too? A. Then I must give it up to you in that sense. — Q. Is it your opinion, at all events, that a mind can be sound and unsound at the same time? A. Except with the qualification I have over and over again stated to you. — Q. Did you state to Dr. Winslow that there was acetate of lead in the milk? A. I did not state that to him. Dr. Winslow must have misunderstood me in conversation. When I saw Dr. Winslow's report, I made an affidavit afterwards to correct that.

Re-examined by Mr. Serjeant Wilkins.—Q. You said just now that there are delusions and insane delusions? A. I did. — Q. And I think you began by making a statement which might have rendered all this vehemence unnecessary. Did you not say something about the Mormonites? A. Yes. — Q. As far as your reasoning enables you to judge, can there be any doubt that the Mormonites labour under a delusion? A. That is certainly my belief. — Q. Would you, therefore, pronounce the Mormonites to be insane? A. No, that could not be done. — Q. There are some gentlemen who believe in clairvoyance, do you believe them to be insane?

A. No; although I believe them to be under a delusion. — Q. There are some people who believe that they have cured patients by means of mesmerism? A. That also is a delusion. — Q. Would you say that all those persons are insane? A. No. — Q. Some years ago there was a sect who made a great noise in this kingdom, who believed that Johanna Southcote was going to give us a Shiloh, do you believe that all the followers of Johanna Southcote were insane? A. No. — Q. Suppose a person to labour under a palpable delusion, and afterwards to reason herself into the belief that it is a delusion, does that show unsoundness of mind? A. That would not indicate unsoundness of mind. Q. Suppose Mrs. Cumming did at one time think that her daughter meant to strangle her, and afterwards stated she was convinced that that impression was erroneous, would that indicate anything like unsoundness of mind? A. On the contrary, I think it would show the integrity of the ordinary faculties of the mind.

Examined by the Commissioner.—Q. You say you do not believe delusions to be insanity, but do you believe them to be tests of insanity? A. Not absolutely, certainly not. — Q. Not tests? A. No, there are many insane persons who have delusions, and others who have not. — Q. What do you consider a test of insanity? A. An obvious impairment of the reasoning faculties, and the other powers of the mind, generally evidenced by other tests. Q. Different circumstances might drive you to the conclusion? A. Yes. — Q. You do not think that the existence of delusions is a test which would drive your mind of necessity to that conclusion? A. Not necessarily, although undoubtedly in many cases they are the indication of insanity. I say there are insane persons who have no delusions, and sane persons who have delusions, therefore delusions cannot be absolutely considered as a test of insanity. Q. Would you draw a distinction between a diseased mind and an unsound mind? A. No, I do not draw that distinction myself, I use the terms synonymously. — Q. If a man's mind is diseased is it unsound? A. Yes.

*Robert James Hale, Esq., M.D.*, sworn, examined by Mr. James.—Q. I believe you are a doctor of medicine, and a licentiate of the College of Physicians? A. I am.—Q. Do you know this lady, who is the subject of inquiry here, Mrs. Cumming? A. I do. — Q. When did you first see her? A. In the latter part of 1847.

A Juryman.—What month? A. It was either November or December. — Q. Last year? A. 1847.

Mr. James.—Q. Upon what occasion did you make your first visit to her? A. I was sent for in a hurry; she was in a fit. — Q. Where was she then residing? A. In the Queen's-road. — Q. Where she is now? A. Where she is now. — Q. When you went there, in what state did you find her; what was the fit? A. I found her on the ground; it was of an epileptic character; very slight. — Q. Did you attend her for some little time? A. I paid her one or two visits, and discontinued my attendance. — Q. Before you discontinued your visits she was perfectly restored to health? A. Yes. — Q. It was merely temporary? A. Yes.

Mr. James.—Now, on this occasion, you say you had not much conversation with her, but was there anything at all about her conduct that struck you as strange or remarkable. A. Not at all. — Q. When did you next see her? A. January the 24th, 1848. — Q. Upon what occasion did you see her then? A. She was suffering then from an affection of the bladder. — Q. She has paralysis, has she not? A. Yes; slightly so. — Q. All that you attended her for was physical infirmities? A. Yes. — Q. How long did you see her then? A. I saw her to February the 23rd. — Q. Did you see her daily? A. Yes; or nearly so. — Q. Had you the opportunity of conversing with her then? A. Yes. — Q. Just state generally; perhaps you had heard at this time, which may have directed your attention to it—had you heard she had been the subject of a Commission in 1846? Did you know that fact at the time? A. Yes. — Q. Then you knew you were attending a lady who had been the subject of inquiry in 1846? A. Yes. — Q. And were you aware of the fact of the Commission having been withdrawn? A. Yes; I was not aware of so much as I am now. — Q. Did that attract your attention more than if you had been called to an ordinary person? A. Yes. — Q. During the whole of that time, from January to February, did you converse with her? A. Yes, I did; in fact, she is a very conversant old lady. — Q. Did you see anything about her at that time to indicate that she was of unsound mind? A. Not at all. — Q. Did she ever at that time talk about her children? A. Oh, yes. — Q. In what way did she speak of her children in 1848? A. I do not know that I can relate any conversations, but she spoke a great deal about her children, the way in which they had acted towards her, and stated that although nominally she had been put into a lunatic asylum by her husband, he was a very old man, and could not be the mover in anything of the kind, and she had to thank her daughters for it. She was always very violent in her expressions against her daughters in consequence. I am not recollecting exactly the words, but the substance of what she said. — Q. Is she an irritable person? A. Very.—Q. Would her diseases physically render the mind more irritable? A. To a certain extent they would; I should say she was naturally so. — Q. And is she a woman who would express herself strongly on any subject. A. Yes; always something superlative. — Q. She spoke of her children in the way in which you have told us, and spoke of their treatment of her? A. Yes.— Q. Upon any other subject was there anything that struck you? A. She spoke of that matter about the milk. — Q. What did she tell you about that? A. She told me at that time that the poison was in the milk, and that she had given it to her cats to drink, and that the cats would not take it, and that roused her suspicions; and the same day, or day after, I forget which, she said a fowl was brought up to her dead; and that Dr. Barnes had analyzed and found poison in the milk. That was her statement then; it is somewhat modified now. — Q. Should you call that an insane delusion if there were any foundation for the fact? A. Not if there was foundation for the fact. — Q. Have you heard Dr. Barnes's statement about the analysis he made? A. I have spoken to Dr. Barnes several times concerning it. — Q. Before you arrived at an opinion that that was an insane delusion, should you not institute an inquiry as to whether there was any foundation, or existence, or supposed existence for the fact? A. Of course; because it would depend upon that whether the delusion was a mere delusion or an insane delusion. — Q. All, or many individuals reason differently from certain premises? A. Certainly. — Q. Some arrive at right and some at wrong conclusions? A. Certainly; the mind is not constituted alike. — Q. Do you, having ascertained the fact, and conversed with Dr. Barnes, consider that to be an insane delusion? A. Certainly not. — Q. Have you given us, as nearly as you can, the general outline of her conversations with you up to that time. A. She used to talk to me sometimes an hour at a time; I had a great many conversations with her. — Q. Loquacity on the part of a lady is not insanity? A. Certainly not; if so, I have a great many insane patients. — Q. Now, as to her cats, did you observe at that time her fondness for her cats? A. Yes; she was very fond of her cats. — Q. You attended her frequently; I presume in her bed-room? A. Always in her bed-room. — Q. What did you observe about her bed-room? A. I observed nothing particular at all. — Q. Was that from January up to February, 1848? A. Yes. — Q. Have you seen sometimes her cats in her bed-room. A. Oh frequently; I have often had to let them in and out of the door; they used to scratch the door when they wanted to come in or go out. Mrs. Cumming has frequently asked me to let them out. — Q. Did you see anything in her bed-room to justify the statement as to the state of filth in which it was? A. No; certainly not. — Q. Nothing of the kind? A. Nothing of the kind.— Q. And you were in her bed-room constantly? A. For a year and a half.— Q. Now, what was the next time you were sent there? A. Next time I was sent for to see her at St. Leonards-on-Sea; that was September 4th, 1848. — Q. The same year? A. The same year. — Q. What was the cause of sending for you then? A. She was suffering then from fever—a little delirium. — Q. A temporary delirium? A. Yes; consequent on fever. — Q. How long did you attend her then? A. I remained with her the whole of that day, and came back in the evening, and I recommended her to remove to Brighton, because St. Leonard's was a very inconvenient distance for me to come and see her; I could not get back to do anything for myself. — Q. When did you next attend her? A. I saw her then at Brighton, in September, 16th and 17th.

A Juryman.—In the same year? A. Yes; the same year; she went from St. Leonard's to Brighton.

Mr. James.—When did you see her again in London? A. Then I saw her occasionally in November and December, in the Queen's-road. — Q. What year? A. The same year. Then I was called up in the middle of the night, on the 1st of January, 1849. — Q. Was she then in the Queen's-road? A. She was then in the Queen's-road; she was then suffering from extensive inflammation of the lungs, and pleurisy; sitting up in bed she could hardly breathe, and I attended her from then to February the 15th. — Q. It may be considered, I fear, a little tedious, but I must ask you these questions. Now, from January 1st to February 15th, did you see her nearly every day? A. Every day; sometimes twice, sometimes three times. — Q. Now, we will ask you, as we have heard evidence upon that, what was the state of her bed-room as to filth? A. Nothing of the kind. — Q. What? A. There was no filth at all. The room felt close, in consequence of her never having the window open; in fact, she was very subject to a chronic affection of the eye. — Q. That is visible now, is it not? A. Yes, the slightest thing affects her.— Q. The room was close? A. Yes; and not only that, but she has bodily infirmities. Q. Was it a fact, that she was labouring under bodily infirmities, which rendered those sort of transactions perfectly involuntary on her part? A. I believe so; I have always looked upon it as so. — Q. You have heard the statements given by some of these servants as to the state of the rooms; as far as you observed it, from January down to February 15th, 1849, is there any ground for such statements? A. I do not believe there is a word of truth in it. — Q. Was there any filth that you observed? A. None. — Q. You say the room was close from the windows not being opened? A. Yes; and from her bodily infirmities. — Q. You observed, I suppose, her partiality for the cats? A. Yes. — Q. Was there anything in it that struck you as strange? A. No, not the slightest. — Q. Do you remember who the servants were? A. No, I do not. — Q. Was she living comfortably and respectably, as far as you observed? A. Yes, always. — Q. Was there at that time anything in her manner, tracing it from January to February the 15th, was there anything in her manner that indicated any unsoundness of mind? A. Quite the contrary. — Q. Will you be kind enough to tell us when you next attended her? A. She had a relapse on March the 10th, and I attended her then till the 27th. — Q. March the 10th in the same year? A. Yes. — Q. The 27th? A. Yes; that was the last of my attendance.— Q. June, 1849? A. 1849. — Q. Pleurisy, was it? A. Inflammation of the lungs and pleurisy. — Q. When did you next see her? A. I saw her then at Brighton, October the 28th, 1851. — Q. Were you sent for? A. Yes, it was a message conveyed in a letter to Mr. Haynes, in which there was a postscript for me to go down immediately to Brighton. — Q. Did you go down? A. I went down by the twelve o'clock train. — Q. Where did you find her? A. In the back drawing-room, which was her bed-room. — Q. In what state of mind did you find her? A. She was exceedingly excited and frightened; in fact, when I was in the room there were one or two knocks at the door, and she started and said, "There now, they will take me to a lunatic asylum." — Q. Have you seen any keepers there? A. Down stairs. — Q. Keepers? A. I could not say they were keepers; I saw four persons there. — Q. Do you know who they are? A. Not of my own knowledge; I was told one was Mrs. Ince, two of them were keepers, and the fourth was, I think, Mr. Turner. — Q. Were they men or women keepers? A. I think it was a man and woman keeper. — Q. Mrs. Ince, a man and woman keeper, and Mr. Turner, the attorney? A. Yes. — Q. You said she was excited and alarmed? A. Yes, of course; I will not vouch who they were. — Q. You found her very much excited and alarmed? A. I did. — Q. What passed; what did you say to her; did she describe to you what had happened? A. Yes; she told me that her door had been broken open violently, and that she had been examined by Sir Alexander Morison and Dr. King and Mr. Turner. She complained very much of the mode in which the examination was conducted. — Q. What did she say about that, as nearly as you can remember? A. She said that a great many very coarse questions were put to her, and some she said she would not answer. To ascertain the truth of this, I asked Watson, who was in the room, what was the nature. — Q. In her presence? A. Yes, in Mrs. Cumming's presence. — Q. What was the nature of the conversation? A. Of the questions. — Q. Well? A. And she did state things to me that somewhat surprised me, and which I had rather not repeat, relative to her husband. — Q. Were they, in your opinion, coarse and indecent questions to put to a lady? A. Mrs. Watson stated to me that they were put by Mr. Turner. — Q. I am afraid we must have what was said? A. I had much rather not repeat the expressions.

A Juryman.—We are not here to try Mr. Turner's conduct. I do not think it has anything to do with the question.

The Commissioner.—How this lady was treated by Mr. Turner may be material.

Mr. James.—Most material, sir.

Witness.—She was asked about her husband, if he was not a very gay man, if he had not connexion with the servants and nurses, and the different persons; to which Mrs. Cumming did not reply.

Q. State it shortly, if you please? A. And then Mr. Turner, so Mrs. Watson said, asked Mrs. Cumming, "Did you not see Captain Cumming do the thing?" Of course, I do not vouch for the truth of this. I have nothing to do with it.

The Commissioner.—Q. You were present when this was told to Mrs. Cumming? A. Yes, there was a good deal in the same kind of strain. — Q. In an offensive and indecent manner? A. Yes. I expressed myself at the time, that it was bad enough for a physician to ask those questions.

A Juryman.—Did she answer while this was going on? A. Yes, she said it was so. — Q. And you say she stated she refused to answer? A. She did. — Q. Now just state as shortly as you can what passed? A. I was in the room for some time, and after that I left with the intention of calling upon Dr. King. I went to his house, but I did not see him. — Q. Did you leave her at Brighton that day? A. No, I returned again, and found Mr. Elliott in the drawing-room with Mr. Haynes, that is the keeper of the Effra Hall Lunatic Asylum. — Q. You gave a certificate that she was not fit to be removed? A. Yes, I did. — Q. And at that time, before you gave that certificate, you saw Mrs. Cumming? A. I did. — Q. Did you believe, at the time you gave that certificate, that she was not fit to be removed? A. Yes.

Mr. Petersdorff.—It had better be put in.

Witness.—I gave that certificate to Mr. Elliott. — Q. I believe you made an affidavit in verification of it. A. I did. — Q. Was she in your opinion at that time in a fit state to be removed? A. Clearly not; and what made me give a certificate more strongly was this, that I was told that she was to be removed by the railroad, and I had heard Mrs. Cumming express herself so many times of her aversion to railroads, that she had never been on one, and she hoped she never should, and I think, if I remember right, there was something of that kind mentioned in the certificate. — Q. You made an affidavit that she was not then in a fit state to be removed as she was? A. Yes. — Q. How long did you remain with her? A. I saw her that evening, and I remained at Brighton that night. — Q. That was the night of the 28th. A. Yes, and I saw her on the morning of the 29th early. — Q. Where? A. On my visit then I saw either one or two persons in the parlour who had remained there all night, I was informed.

Mr. James.—Q. Who were they? A. I was told they were the keepers from the asylum? Q. Were they the same people you had seen before? A. I imagine they were. — Q. Did you see Mrs. Cumming? A. Yes, I did. — Q. In what state did you find her on the 29th. A. I found her rather quieter than she was the day before, but she was in great fear, she knew that directly I went to London they would take her off to an asylum. I said what I could to pacify her. I stated they would not do anything of the kind. — Q. You did what you could to pacify her? A. I did. — Q. Did you then leave Brighton? A. I left Brighton. I made an affidavit the same day in London, and I did not see her again till November the 26th, after her removal from the asylum? — Q. Where did you see her? A In the Queen's-road, where she is now. — Q. After she had been taken to the Asylum at Effra Hall, and brought back to the Queen's-road, you saw her on the 26th of November. A. Yes. — Q. Have you seen her constantly since? A. I have seen her about three times a week since. — Q. Up to the present time? A. Yes. — Q. You have had many opportunities of seeing her and ascertaining the state of her mind? A. Yes, I have. — Q. Is it your opinion that she is of sound or unsound mind? A. It is my opinion that she is of sound mind. — Q. I will first ask this question as to her physical state on the 26th of November. Did you find her then altered after she had been at the Effra Hall? A. In fact she was suffering greatly from exhaustion, she could really answer no question whatever. —Q. What day had she come from the asylum? A. I do not know, she had been removed some few days. — Q. She was very much altered? A. Yes. — Q. Suffering greatly from exhaustion and could hardly answer you? A. Yes. — Q. Was she suffering pain did she say? A. Yes, great pain in her bowels. I think she had diarrhœa. — Q. And nervousness? A. Very much. — Q. And her nerves had been shaken? A. Very much; in fact, to use her own expression, she wished that she might be left to die. — Q. That she stated to you when you saw her when she first came from the asylum? A. That was the first visit; I said very little to her.

Cross-examined by Mr. PETERSDORFF.—You say that you first knew Mrs. Cumming in 1847? A. I did. — Q. Where were you yourself residing at that time? A. I resided—in fact, I had been only in St. John's Wood about a month, in the Queen's-road. — Q. Were you living near Mrs. Cumming? A. About twenty yards off, perhaps. — Q. From her present residence? A. Yes; immediately opposite. — Q. Was your first knowledge of Mrs. Cumming derived from her accidentally sending to you? A. Yes. — Q. You had no introduction at all? A. No. — Q. Were you at the time acquainted with Mr. Haynes? A. No, I did not know Mr. Haynes for a month afterwards. — Q. Have you directed much of your attention to cases of insanity? A. I have seen a great deal of it. — Q. What opportunity have you had of seeing a great deal? A. I was a pupil of the celebrated Dr. Pritchard, of Bristol, and attended Saint Peter's Hospital, where there are several wards devoted to insane cases. — Q. Then, as I understand you, your knowledge of insane cases was derived from the information you got during your pupilage? A. No; I have had several cases, and even now. — Q. How many cases do you think you have had under your own superintendence? A. Really, I cannot say. — Q. How long have you been in practice on your own account? A. About twelve years. — Q. And have you had about thirty patients? A. Yes, private patients. — Q. Have you no public institution under your arrangement? A. Yes, I have; I am physician to the Western General Dispensary, not an insane institution. — Q. You are not connected with any public institution for the reception of the insane? A. No. — Q. You have stated that a number of times you have seen Mrs. Cumming, and given us a long list of dates. Will you tell me whether during the time you were attending her there was any other medical attendant besides yourself? A. Not perhaps at the precise time I was attending. — Q. But about those times? A. Yes. — Q. Can you fix about the time there were other medical persons attending the patient besides yourself? A. Dr. Caldwell, I imagine, attended her in 1848, from about March to about August or September. — Q. Were you in the habit of meeting in consultation on this occasion? A. No, not at all. — Q. Can you give me other times at which you know Dr. Caldwell or some other medical man was attending as well as yourself? A. I believe Mrs. Cumming was exceedingly fond of having medical gentlemen. I am sure she has had enough of it lately. — Q. Will you be kind enough to tell me if you can fix on other times when other medical men were attending her as well as yourself? A. I imagine Dr. Caldwell might have attended her after September perhaps, to November or so; I think she has told me she had seen Dr. Caldwell, she made no secret of it. — Q. You have said that you attended very frequently indeed upon Mrs. Cumming—about what time used you to go? A. At all times. — Q. Were you ever there early in the morning? A. I have been there very early in the morning—six o'clock in the morning, when she has sent for me. — Q. What was the time of your ordinary visits? A. About eleven or twelve. — Q. I suppose at those times when you called it was an understood thing that you would make your visits about eleven or twelve? A. Yes. — Q. When you went at eleven or twelve in the day the room was to rights? A. Yes, exactly. — Q. Did you at any time visit Mrs. Cumming on the footing of an acquaintance, or was it always professional visits you paid her. A. I used to see her often upon the footing of an acquaintance; frequently I called without expecting any fee at all. — Q. Have you ever dined with her, or drank tea? A. I think I have dined with her once. — Q. In the course of all these visits, did not Mrs. Cumming frequently introduce the names of her daughters? A. Frequently. — Q. Was not that a prevailing topic when she had these conversations for an hour or two hours together, and she was very chatty? A. It was not what I could call a universal topic. — Q. Was it not a very prominent topic in her conversation? A. Sometimes it was, but not always. — Q. Will you, then, be kind enough to tell us whether you have not heard Mrs. Cumming, over and over again, express the strongest feelings of prejudice against her daughters? A. I have heard her express very frequently, and in very strong terms, her feelings against her daughters, in consequence of things that had occurred. Q. Did you ever hear her adopt at any other time a manner except that of very strong prejudice and dislike. A. I think her remarks, generally speaking, were directed against the husbands of her daughters. — Q. Did you ever hear her assign, for instance, any reason against Mr. Ince for her detestation of him? A. The reason that she assigned for her disliking Mr. Ince, that she believed he had been, in a sense, instrumental in persuading her daughters to take proceedings against her and putting her in a lunatic asylum—that was the notion she had—and against Mr. Hooper, she disliked him because he was a trumpeter, she called him. — Q. In these conversations, when she mentioned the name of Mr. Hooper, did she always persevere in charging him with being a trumpeter.

Mr. James.—He was a trumpeter.

Mr. Petersdorff.—At the time you heard Mr. Hooper's name mentioned, was not her complaint against Mr. Hooper that he was a trumpeter? A. That was one of her complaints. — Q. Do you remember any other complaint she brought against Mr. Hooper? A. That she thought it was a very great degradation for her daughter to marry him. I need not tell you Mrs. Cumming is a Welsh woman. — Q. What inference we are to draw from that I do not know?

Sir Frederick Thesiger.—Because they are fond of their pedigrees.

Mr. Petersdorff.—Did she boast at all of her exalted descent? A. She was very proud. — Q. Was there any other ground of complaint against Mr. Hooper than as being a trumpeter? A. A trumpeter, and being in station beneath her daughter. — Q. In these conversations, did you hear from Mrs. Cumming that she had been perfectly reconciled to Mr. and Mrs. Hooper for several years? A. I think I did hear something of the kind. — Q. Having heard that they were reunited, did Mrs. Cumming at any time suggest to you any reason why she renewed her feelings of hatred against Mr. Hooper? A. Not that I remember. I do not charge my memory with it. — Q. Did you not learn from Mrs. Cumming that the marriage of Mrs. Ince was with her perfect consent and approbation, and the consent and approbation of Captain Cumming? A. I really do not know. I should not like to answer. — Q. You have said that Mrs. Cumming was a woman of an irritable temper, or only at times? A. No; very affable occasionally. — Q. When she became irritable did you observe that there was any reason for her change of manner? A. I think she generally used to get so from her own description of her wrongs. — Q. Am I to understand she worked herself up in a passion in that way? A. Yes, frequently. — Q. When she talked about this proceeding of the milk—you say she mentioned that very often—did she say who she thought had introduced the poison? A. No, I do not know that she ever showed it to me. — Q. Will you undertake to say that she did not state who it was that she suspected of having poisoned the milk? A. I will say that I do not recollect Mrs. Cumming saying to me who she thought it was. — Q. Will you swear she never expressed to you her suspicions on that subject? A. Certainly I will. — Q. You represent to the jury she never on any occasion at all intimated to you whom she supposed poisoned the milk? A. I do not believe she ever did. She stated to me often that there was poison in the milk, and that her fowls died. — Q. Did she ever suggest to you how she thought the poison had been introduced into the milk? A. No. — Q. Did you never ask her? A. Not that I know of. I may; but I do not remember. — Q. Will you undertake to swear that when she made that statement about the poison in the milk, your information did not suggest questions to her? A. I should be sorry to say at this distance of time. I did not ask the question at the time; but my impression is, that she never did state to me who she suspected, most certainly. — Q. You have stated your opinion that Mrs. Cumming is of sound mind. Will you have the goodness to explain what is your definition of a sound mind, as contrasted with an unsound one? A. I take it, a person is of sound mind whose conduct, thoughts, actions, and affections, are in accordance with those of the great mass of mankind. As regards the standard, it is a very uncertain thing to talk of a standard of unsoundness, because we generally take ourselves as the standard, which is rather a fallacious thing, so that I should not take a person of unsound mind who differed from myself. — Q. You would take as a test of perfect soundness of mind, your own natural mental capacity? A. I would. — Q. Now supposing you were to labour under some delusion, not as a theory or speculative doctrine, but with respect to a physical fact, and that no reasoning or evidence could remove the erroneous belief from your mind, would you say that was indicative of soundness of mind, or the opposite? A. There are many delusions. — Q. Answer the question, if you please?

Mr. James.—He must finish his answer.

Sir Frederick Thesiger.—It was perfectly clear that he was not about to give an answer to my learned friend's question.

Mr. James.—He may give an answer, and if it is not an answer, he may repeat the question.

Witness.—I never answered it.

The Commissioner.—Put the question again.

Mr. Petersdorff.—I take your mental capacity as a test of soundness? A. You may, if you like. — Q. I dare say we cannot have a better. Supposing you believed the existence of a physical fact which was shown to you by demonstrative evidence did not and could not exist, would you say that a permanent belief in its existence was consistent with that soundness of mind you perhaps justly attribute to yourself? A. It might or might not, under particular circumstances, according to what that fact was. If you will mention what it is, I will tell you; there are so many physical facts. — Q. I will illustrate what I mean. Suppose you laboured under a delusion that the table at which those gentlemen are sitting is not a table, and you persevered, year after year, in defiance of the physical evidence of the existence of the table, that it was not a table, would you say that was indicative of insanity or not? A. A person who believed that which was acknowledged by all the world to be a table, and who could not be reasoned out of it, that would be a delusion. — Q. And it would be evidence of insanity? A. It would be an insane delusion. — Q. Supposing you were reasoned out of the erroneous belief as to that not being a table, and after the lapse of a year or two, were to return again to that delusion, would you say that that was indicative of returning insanity? A. It frequently is the case in insane cases. — Q. As to the test of insanity, is there not a distinction between a disbelief in an ascertained physical fact, and a disbelief with respect to a mere theory or doctrine? A. Yes, there is a difference. — Q. With respect to a want of evidence as to a theory or doctrine, would you say that was indicative of insanity? A. Certainly not. — Q. But if it related to a positive doctrinal fact, it would be? A. I imagine it would. — Q. You have obliged the jury, though it was rather an incidental remark, as to the distinction between delusions and insane delusions? A. Yes. — Q. Will you point out to the jury the difference between a delusion, and a delusion which constitutes insanity, according to your notion? A. I take it, an insane delusion is the expression as to the reality of things which do not really exist and the action of the person in accordance with that belief, or you may have an insane delusion where there is some foundation in fact, but the ideas are carried out to an absurd and extravagant extent: those are insane delusions.

Mr. Petersdorff.—Q. I suppose you would not call it an absurd delusion for a person to keep two or three cats? A. Certainly not. — Q. But would you call it an insane delusion if some person kept five or six cats, kept them in her bed-room, and scarcely ever allowed the door to be opened?

The Commissioner.—Q. Is keeping cats a delusion? A. Certainly not.

Mr. James.—The keeping a live cat cannot be a delusion.

Re-examined by Mr. James.—Q. Keeping a cat, or five thousand cats is not a delusion? A. No. — Q. And that would rather range under an eccentricity bordering on extravagance of mind? A. Yes. — Q. I asked you whether you think keeping six cats is such an extravagance as would be a test of insanity? A. Certainly not. — Q. My learned friend put Bishop Berkeley's theory, the belief of a table not being a table. I suppose if a person stated to you that a table, which you yourself thought was a table, looked like a sofa, before you pronounced that it was a delusion, you would inquire whether there was any disease in the eye? A. Yes. — Q. If a person whom you were called on to attend, said, "That table is not a table, it appears to me to be a sofa," would not the first thing you would inquire be, whether the eye was in a healthy state? A. No doubt there are many speculative delusions. — Q. If a person told you it was not a chair, but it was a stool, would not your first question be to inquire what was the state of the retina of the eye? A. No doubt.—Q. Must it not therefore depend entirely upon circumstances? A. No; every case must be judged by its own merits. — Q. Another question was put to you, which a little unintentionally and unfairly represented your answer. I think you said that an examining party called in to test insanity, naturally takes as the standard of sanity his own case? A. Of course. — Q. You do not set up your own state of mind as the standard of sanity, but as a standard of sanity by which the examining party tests the examined party? A. Yes. — Q. Perhaps the same things must be said of a jury? A. Yes. — Q. And to some extent, therefore, you set up the standard which exists in your own mind, of what seems to be, to you, to be sound? A. Yes; but I stated that that must be a fallacious standard. — Q. You have given a definition of delusions which are insane, there are delusions which are not insane, are there not? A. Certainly. — Q. Will you give us an instance, if you please, of a delusion which you do not believe to be insane? A. Speaking professionally, I should say that I believe mesmerism to be a delusion—

Sir Frederick Thesiger.—That is not a fact, but a theory.

Mr. James.—Q. May not that theory be so strongly existing in the mind of a person as to be what is properly called a delusion? A. Certainly. — Q. I believe the mesmerites alleged it to be a theory supported by facts? A. Certainly. — Q. And you believe it to be wrong. A. Certainly; I do not believe persons who believe in mesmerism to be insane; there are many sects in the world, too, that suffer from delusion. —Q. You have been asked as to a disbelief of an ascertained fact, that must depend, must it not, very much on circumstances? A. Certainly.

A Juryman.—I do not understand what you said about the next generation, that would be disproved in the next, consequently they are no longer facts.—Q. Would you believe a person mad whom you could not convince that George the First lived historically? A. Certainly not. Speaking about facts, there are many things now stated as facts, which, by further experience, will be proved to have exceptions to them, and a thing which is an exception cannot be a fact.

Sir F. Thesiger.—That is the progress of science? A. Exactly.

Mr. James.—You were to put a general question. Would a permanent disbelief in, and ascertained, be an insane delusion, and your answer was, it might, or it might not. Must it not depend upon the ascertainment of the fact, and the agreements you bring to the mind of the individual for its existence?

The Commissioner.—So long as the fact remains a fact, the inference to be drawn from it must be the same? A. Yes.

Mr. James.—And you would make a great distinction between the disbelief, in fact, permanently brought to the mind of the individual, of the existing means on which she had more evidence of its existence. For instance, suppose a person who did not believe that the "Amazon" was lost the other day, would you believe that person was mad? A. No. — Q. Should you say Joshua was mad because he demanded the sun to stand still? A. No. — Q. Must it not depend upon the ascertainment of the fact as brought to the mind of the person? A. Certainly — Q. Would you say a person was mad who would not believe that the "Amazon" was lost? A. No. — Q. But if that person saw the vessel go down, and would not believe it was lost, you would? A. Yes. — Q. Must it not depend upon the existence of the fact, as brought to the mind of the individual? A. Certainly. — Q. Did you frequently call upon her without her knowing you were coming? A. Yes, at all times. — Q. So that you found her in her bed-room without any particular care to receive you? A. Yes, I have been there as late as ten or eleven. — Q. And at six in the morning? A. Yes; I never went at six or seven, unless I was sent for. — Q. Have you been at all times? A. Yes, at all times.

A Juryman.—In all your conversations with this lady did you ever have any conversations about her property? Yes. — Q. Will you state what she said to you about her property? A. She has merely told me that she had estates in Wales. — Q. Did she ever tell you about selling any of her property? A. Yes; she told me she had sold three or four places, and about the railroad. — Q. Did she say what she sold them for? A. I do not know that she mentioned the amount. — Q. Did you not ask her what the railroad gave her? A. I think I have asked her lately. — Q. Did she ever tell you the amount she got from the railway and water-works. A. I think about £6000. — Q. For the four? A. Yes. — Q. Did she say how much for each? A. I think Bassaleg she said 2000*l.* There were two and 2000*l.* and 3000*l.* or a little more each. — Q. Did she ever talk about a will? A. Yes; she often told me she had made a will, but she had never signed it. — Q. Did she tell you when she made the will? A. It was a very long time ago—I think it was about, or shortly after, the former commission. — Q. After the commission she spoke about having made a will? A. Yes. — Q. Did she positively tell you she had never signed it? A. No. — Q. Did she tell you she had ever burnt a will? A. No; I think not. She told me she had not left her daughters anything. — Q. Did she tell you who she had left her property to? A. She said she had left her property to those people who stood by her in the former commission. I had often spoke to her about her living, as I termed it, so very fast; but she said she never intended to leave any money behind, so that there should be no litigation about it. She always had the determination not to leave any money; she said she never would. — Q. On the 24th September you attended this lady at Hastings? A. September 4th at St. Leonards. — Q. And there was delirium tremens? A. Yes, she was suffering from fever. — Q. Was it delirium tremens she had at that period? A. I can only go by hearsay. I was told that that fever was brought on by her drinking a little too much wine or brandy, or something of that sort.

The Commissioner.—Do you mean that you made the inquiry when you were down there? A. Yes. — Q. So as to satisfy your mind as a professional man? A. Yes. Before you could begin any treatment you must find out what is the matter with your patient.

A Juryman.—Since that period you have attended her, have you had any reason to believe she was under a similar influence? A. I have not seen her what you would term intoxicated, but I believe that I have seen her some few times when, perhaps, she has taken a little more wine or brandy than was of service to her—nothing further than that. — Q. Do you know what quantity of wine or brandy she was in the habit of taking in the course of the day. A. I think she takes some few glasses of wine. — Q. What quantity of wine? A. I should not think a third of a bottle, or half a bottle, at least. — Q. What quantity of brandy? A. I imagine at that time it was a couple of glasses. — Q. In the course of the day? A. Brandy and water I am speaking of. — Q. Do you suppose a person in her delicate state of health taking that quantity of wine and brandy, that it would produce the effect of delirium tremens? A. Yes, I think so; more especially as Mrs. Cumming's digestion very frequently is very bad, and frequently she eats very little, consequently. — Q. But, by taking the quantity you have alluded to, it might have, up to the present time, the effect of causing a derangement of her intellect? A. I would not say that. I think it may account in a great measure for her burst of passion. I think that is very possible.

The Commissioner.—You think the quantity of wine, or brandy and water, might account for it? A. For her passion. I do not exactly understand you. — Q. You said something might account for these ebullitions of passion? A. I imagine that all persons who take little alcoholic stimulants makes them rather irritable.—Q. The nurse says she takes a bottle of port in a day and a half; is that too much? A. It is a very large quantity; I should not like to take it myself.

The Commissioner.—Would you recommend it for her? A. No; certainly not. There are cases I know of. A lady I attend, with a member of the College of Physicians, and the lady took nothing but gin, and I think she took a quart of gin daily; and as regards anything solid, there was not half an ounce of food taken, and she lived for six weeks upon it, and it was the thing that sustained life.

A Juryman.—Would not taking that quantity of gin produce continued weakness? A. It would eventually exhaust the powers of life; there is no question about that; but it sustained life for a time.

The Commissioner.—You gave a certificate that she ought not to be removed from Brighton? A. I did. — Q. She was removed? A. Yes. — Q. Were you surprised that she suffered no material injury from it? A. I was very much surprised.

A Juryman.—Did you ever hear her state, out of the money she received from the sale of her property she had paid for law expenses? A. I think she has told me, two or three thousand pound the inquiry cost. — Q. You were never told it was £5000? A. No; I am speaking merely of the Commission. — Q. I am speaking of the money she has received from her estates, how much money she has paid for law expenses? A. What I was stating was merely the commission, to which she said there were law expenses since.

The Commissioner.—She told me she had 2000*l.* to give her daughters. A compromise that she had given her daughters 2000*l.*, and that she abused Mr. Haynes very much for it at the time. She said he was only making bullets for her daughters to fire at her. You have heard her abuse Mr. Haynes? A. She abused Mr. Haynes for it. — Q. Did you ever hear her abuse him for anything else? A. Really I do not know—I may or may not. I do not remember the circumstance.

Q. You seem to have seen her in different kinds of illness. Do you think her memory is still affected? A. Now? — Q. Yes. A. It is not the same as when I attended; it is very much impaired lately. — Q. Her mind? A. No, her memory is very much impaired. — Q. Since when do you think this has been? A. The last time I saw her was March the 27th, 1849, except lately. — Q. Are there not other circumstances that lead you to the conclusion that a person is in a wrong or right mind. A. Certainly. — Q. A question of fact. A. Yes. — Q. Did you ever see any person have strong opinions with reference to their children, who were considered to be in their right mind? A. Provided there is no cause. — Q. No original foundation? A. No original foundation. — Q. Supposing there to have been a good original foundation for that unfortunate hatred, and you find your patient becomes suspicious, and applies suspicion to everything unconnected with those same individuals, is that any symptom at all, I will not say a test, of sanity or insanity? A. I do not understand the question. — Q. Suppose a person has a great hatred against her children? A. Without a cause. — Q. Without a cause; and you find that take such possession of the mind that other people are looked on with a jealous eye, and supposed to be in connivance with those daughters. A. A morbid perversion of the affections no doubt is an unsound state of mind; morbid perversion of the affections I am stating, of course, without foundation. — Q. Suppose Mrs. Cumming has an aversion to her children without foundation; then she sees them, and is on good terms with them, and on a sudden, without real cause, she takes an impression that other persons are connected with her children,—is that any symptom of insanity? A. It is not. Her mind is so engrossed with the notion that the daughters are persecuting her, and that originates in actual facts and not assumed. I think that the mind of a person so constituted may be induced to regard other persons whom she may imagine friends of these parties with suspicion. — Q. If you can lead a mind on under such circumstances? A. I hardly think if you could, that it would be sufficient grounds for saying that person was unsound. — Q. Would it be, to a certain extent, a test. A. It would lead one to pause; and then, if you had other circumstances to back it, it would strengthen you in your opinion. — Q. You must have many things which draw you to the conclusion—one would not satisfy you.

A Juryman.—If you were called upon to ascertain whether Mrs. Cumming was of sound or unsound mind, you would question her as you have done, in order to come to that conclusion? A. I would put as many questions as I possibly could.

The Commissioner.—When did you first observe her memory fail? A. I only had an opportunity of seeing her since her return from Shoreham. — Q. When? A. In November. — Q. Did you observe any failure in her memory about November? A. I have not seen her since November, 1849, not once; I have never seen her from March 27th, 1849, till she removed from Shoreham, in November, 1851. — Q. You were sent for to Brighton? A. Yes. — Q. Did you observe any alteration in her memory then? A. I had not an opportunity; I was only sent down for a specific purpose. — Q. When you first saw her, after her return to Queen's Road, was it the first time you saw an alteration in her? A. Yes.

A Juryman.—Did you ever, in your experience, see a similar case where a mother, whose feelings were so strong on such a subject? A. Speaking from my own personal experience, I do not think I ever did.

The Commissioner.—If you had been asked when you saw her come home from Effra Hall, to have attested a will, in which she had given her property from her relations, would you have assented or dissented? A. She was not in a fit state to do anything then; she was a person suffering from extreme exhaustion, and, in fact, I could hardly get any words from her at all. — Q. You say she has improved since? A. Very much improved. — Q. When did you see her last? A. I saw her yesterday. — Q. How long were you with her? A. I saw her yesterday, twice. In the morning. — Q. What time? A. About—I think it must have been about ten or eleven o'clock; no, by the bye, she sent for me at nine o'clock. I saw her about nine o'clock, and afterwards went with Dr. Caldwell to see her; but that was merely because she was suffering from diarrhœa.

A Juryman.—If the Commissioner questioned Mrs. Cumming as to her knowledge of what property she had sold, and if on the first day she told us that two lots had been sold for 2000*l.*, and a third for 3000*l.*, and called those three lots 9000*l.* instead of 7000*l.*, and if on the second day she was questioned, and then told him there had been two lots 3000*l.*, and never mentioned the other two, would you draw the inference that she was capable of judging of her property. A. I should draw the inference that her memory was not correct; loss of memory is no test of insanity, especially as she is seventy-six years of age.

The COMMISSIONER.—A total loss of memory may, or may not be? A. Yes. — Q. A total loss of memory is a defect of mind? A. You cannot have a perfect mind with perfect loss of memory; only a great many things are to be said with regard to the age of the person. Now I know several relatives of mine, not so old as Mrs. Cumming, and have not the slightest idea of their property, or very little memory; but I do not imagine I should be justified in saying they are of unsound mind. — Q. Would you allow those persons to make a will with your sanction? A. Not unless it was thoroughly explained; otherwise, if they are not allowed to make a will, you would deprive a number of persons from the just exercise of their rights. — Q. It requires very great caution? A. Yes; no doubt it requires great caution.

*Walter John Bryant*, sworn. Examined by Mr. JAMES.—I believe you are a member of the Royal College of Surgeons? A. I am. — Q. In the year 1846, while the commission was pending against Mrs. Cumming, did you go over to the Horns Tavern and see her? A. I did. — Q. I believe the first time you saw her was in the room while the inquiry was going on? A. I was sitting beside her in the room. — Q. I believe you were taken over there by Mr. Haynes. I believe Mr. Robinson is a patient of yours, his partner? A. Yes; I received a letter from Mr. Robinson, desiring me to go. — Q. Had you then conversations with her? A. I had several conversations with her. — Q. Did you form any opinion of her soundness or unsoundness of mind? A. I formed an opinion that she was of sound mind. — Q. And I believe you were prepared to give evidence of that kind if it was required? A. I was. — Q. When did you again see her? A. I saw her again on the 25th of December last. — Q. Where did you see her? A. At her house in Queen's Road, St. John's Wood. — Q. The same house as she is in now? A. I believe so. — Q. You learned, of course, at that time, that she had been to the asylum at Effra Hall? A. She had come from an asylum. — Q. When did you see her alone? A. I saw her on the 6th of January. — Q. I believe you wished to see her alone? A. I wished to see her alone. Q. Had you conversation with her with a view of forming your judgment as far as you could of the sanity, or soundness or unsoundness of her mind, on the 6th of January, 1852? A. On that day I merely had conversation with her in regard to her property, and on other matters. — Q. Do you remember, in the conversations you had with her, saying anything about her children? A. I told her it was alleged that she had an unfounded antipathy towards her children. — Q. What did she say? A. She said it could not be expected that she could entertain that feeling of affection towards her children she had hitherto done, as they had, on more than one occasion, placed her in a lunatic asylum, and they still intended to do so, she believed. — Q. Did she allude to one child only, or to both, generally? A. She spoke of her children. — Q. Do you remember anything occurring about the poison, the analysis that Dr. Barnes made; I may perhaps remind you you had seen the report which had been made by Dr. Monro and Sir Alexander Morison to the Chancellor? A. No, I have not; I have heard these allegations, and I questioned her upon them. — Q. Now, about the poison and analysis? A. I said it was alleged she had stated some poison had been placed in some milk, and I wished to know if that were true. — Q. What did you say to her? A. That was my question to her; her answer was, that her suspicion had been excited by the fact, that a cat, or cats, had refused to drink some milk placed before it; that about the same time a fowl had died—a white fowl, I believe she said had died—and that she was suspicious that poison had been used for that purpose; that a servant had brought in a paper containing a substance which, together with the milk, she had given to Dr. Barnes, who had analysed the milk and the paper, and had pronounced that it contained poison. — Q. Did you afterwards ascertain whether that was the fact, or did you know before that Dr. Barnes had made some analysis? A. I did not know that he had; she also mentioned some chemist, but at this moment I cannot recollect the chemist's name. Some chemist had assisted Dr. Barnes in the analysis. — Q. Do you remember her saying anything about strangling by Mrs. Ince? A. I then said it was alleged that her daughter, Mrs. Ince, she had said, attempted to strangle her. —Q. What did she say upon that? A. She said it was a falsity. — Q. Did she give you any description of what had occurred, and what was the foundation for the statement? A. She did; I asked her if there was any foundation for such an imputation. She then said, that for some time previously she had been living in apprehension that her daughters intended to place her in a lunatic asylum; in fact, that she believed it was their intention to do so; and that, on one occasion, Mrs. Ince rushed, unannounced, into her room; pushing the servant rudely on one side, she threw her arms about her neck. — Q. Mrs. Cumming's neck? A. Mrs. Ince threw her arms round Mrs. Cumming's neck. She exclaimed at the moment, "Are you going to strangle me?" or some such expression as that. — Q. She says she did? A. She says she did; but recovering from the temporary or transient fear which she experienced by the suddenness and abruptness of the act, she dismissed the impression as quickly. — Q. She told you so? A. Yes, that is the substance of what she said; I do not mean to say that is the precise phraseology. — Q. Did you say anything to her at that time about its being alleged that she had attempted with a knife to make some attack on one of the Hickeys? A. Yes; but previous to that I said, it is also alleged you have attempted to cut the throat of one of your servants. — Q. One of the Hickeys? A. I believe it was Ann Hickey. —Q. What did she say to that? A. Her answer to that was, "It is a falsity. I have been brought up to fear God, and with principles far different to that, sir." — Q. That is what she said? A. Those are her precise words. — Q. Did you ask her who the medical men were who had visited her on that day? A. On the 6th I did. — Q. Whom did she tell you had visited her on the 6th? A. She informed me that Dr. Forbes Winslow and Dr. Monro had seen her. — Q. That day? A. That day. She expressed herself as feeling ill, and feeling exhausted from having seen medical men previously to my visit at that time; she was partaking of some refreshment.—Q. Do you remember anything that she said to you about Dr. Monro? A. She said Dr. Monro had, on his introduction. expressed himself to the effect that she was not to feel alarmed, that he came there in a friendly way towards her; to which she replied, "I can scarcely consider that to be the case, Dr. Monro, as I believe you have signed an affidavit certifying that I am of unsound mind." — Q. During the conversation you had with her, from seeing her on these occasions, did you form an impression as to whether she was of sound or unsound mind? A. From the conversations I had with her on those two occasions, I am certainly of opinion that she is of sound mind. — Q. You have been made aware of the facts, of course, of the previous commission against her, and her being confined in the lunatic asylum? A. I was aware of it. — Q. Although she may entertain very strong feelings of aversion against her children, do you call that a delusion, if there is any foundation in fact for the aversion she has of their conduct? A. It could not be a delusion if there is a foundation for it. — Q. The mere fact of the aversion is not evidence of insanity? A. No, it is a question of degree. — Q. Having ascertained there was some ground for the statement of the poison, are you of opinion that is a delusion? A. No, it is not a delusion. — Q. From the judgment you formed, you believe her to be of sound mind? A. I do. — Q. Her memory is impaired to some extent from age, is it not? A. I could have no opportunity of positively testing that. She did not immediately recognise me as having seen her in 1846. — Q. She did afterwards? A. Yes, she knew me upon that occasion; the light was glancing into the room, and she was evidently suffering from some affection of the eye. — Q. After you had conversed with her for some time, did she know you? A. Not immediately; but she soon recalled to my mind facts that had taken place when I saw her in 1846.

Cross-examined by Sir F. Thesiger.—Q. Have you considered from that that her memory was not impaired? A. I could not say as to the amount of impairment. — Q. You say, on the 28th December, I have had conversations with her; I do not understand whether that was in the presence of Mr. Haynes or not? A. Mr. Haynes was present. — Q. The whole time? A. The whole time. — Q. How long did that last? A. I should think about an hour—quite an hour. — Q. What were the particular subjects which were conversed upon when Mr.

Haynes was there? A. Upon the different allegations as to the attempt of Mrs. Ince to strangle her,—upon her unfounded antipathy to her daughters,—upon the allegation that she had attempted to commit suicide,—and also to cut the throat of one of her servants—and about the poison. — Q. Did you converse with her about the same topics on the second occasion? A. No, I did not. — Q. What were the topics on the second occasion? A. They were as to the property. — Q. Nothing else? A. Nothing else—when I say nothing else, I might recollect some things, but my principal object in my second visit was with regard to her property. — Q. What did she say about her property? A. I asked her what her income was derived from? she answered, from the funds and from lands and houses. — Q. Did you ask her what amount of property she had? A. I did not ask her the amount of property; but the question I asked her, "What do you think is the amount of the annual income arising from this property? — Q. What did she say?" A. She said between £400 and £500 a year. — Q. I want to know whether she represented to you that she had the notion of her daughter intending to strangle her when she came into the room in that way, but that the impression was almost immediately removed? A. No, she did not say that; she said, it was in consequence of her daughter throwing her arms round her neck that the notion was created. — Q. Do you mean she said the impression was removed during the visit of her daughter, or that subsequently it was removed? A. "She reflected," she said, "on recovering from my momentary fear,"—those were her words,—she dismissed the impression that the daughter was about to strangle her.—Q. At the time? A. At the time.

Sir F. Thesiger.—In answer to my learned friend, you have given a sort of definition of a delusion, and you say that where there is any ground, any foundation for it, you do not consider that a strong aversion is evidence of a delusion? A. If there be a ground for it. — Q. But I want to know to what extent you carry that opinion; suppose, for instance, the daughters of a mother had been ungrateful to her, and she afterwards entertains the notion that they are going to murder her, do you consider that the mere circumstance of their ingratitude is a justification, if I may use the expression, for an opinion she entertains and carries to that extent? A. I should not call that a delusion. — Q. Then you think any feeling, however slight, would be sufficient to justify any opinion, however strong? A. No, I could not give an opinion, it must be regulated by other facts. I should have more evidence. — Q. As you have given us the character of a delusion? A. I answer it by saying I do not think it a delusion. — Q. Though there is not the slightest ground for it? A. I understand there was a foundation. — Q. Not a ground for a belief they were going to murder her? A. No, but there was a strong antipathy. — Q. Do you consider the belief of ingratitude would be a sufficient justification of the belief that they were going to murder her, so as to take that out of the character of a delusion. I put it thus—I assume there is a foundation for the opinion of the ingratitude of the daughters, and I want to know whether you consider that that would justify the belief, as a rational belief, that they were going to murder her? A. I could not certainly say it was a rational belief, but I should not like to call it to the extent of a delusion. — Q. I do not quite understand you; what do you consider a delusion? A. A delusion, I should conceive, was the existence of something in the imagination of a person, that did not exist in the fact, that only existed in the imagination. I should not like to be so bold as to give a definition of a delusion, that is my expression. Gentlemen who have paid more attention to diseases of the mind than I have would be better able to answer that question; but that would be my own view. — Q. Do you agree with Dr. Hale, for instance, that if a belief or opinion is very much exaggerated beyond what the truth of the fact warrants, that that would amount to a delusion?

Mr. James.—His expression was to an absurd degree.

*Witness.*—I should not consider that a delusion.

Sir F. Thesiger.—To an absurd and extravagant degree?

A. I should not consider to an absurd degree a delusion—it is a question of degree, that is. — Q. Suppose, for instance, that in some commercial transaction a person had been over-reached; was he in a sound state of mind if he believed that the other had picked his pocket as he was passing through the street? A. Yes. — Q. You would say that was rational? A. I should not say it was.

Re-examined by Mr. James.—Q. Supposing a person narrating a commercial transaction in which he had been over-reached, should you think that a delusion? A. No. — Q. Supposing, narrating the transaction, he had told you I had picked his pocket, should you say that was a delusion? A. No. — Q. You have been asked about her daughters going to murder her—supposing this lady had said that her daughters entertained such a feeling to her, that they would murder her, should you think that a delusion, or a mode of expression as to her opinion of her conduct to her? A. If the daughters had expressed that they would murder her. — Q. Supposing, Mrs. Cumming in describing the conduct of her daughter, that she entertained such a dislike to her that they would murder her, should you consider that a delusion or mode of description? A. A mode of description. — Q. Suppose she said she entertained a feeling towards her that they would poison her, should you think that a mode of description or a delusion? A. A mode of description. — Q. Supposing she said her daughters entertained a feeling that they would not hesitate to murder her, or would murder her, should you consider that very different from the assertion that they were going to murder her? A. Certainly. — Q. Do you consider that a delusion if there is a foundation for it? A. It is a question of degree. — Q. You have stated you have not turned your entire attention to this study, but you have had the ordinary experience of a person in very considerable practice as a general practitioner? A. I should say I have had a fair average.

A Juryman.—The interview you had with her was so recent as the evening before this commission? A. The very evening before. — Q. At that time, in your opinion, do you say she was of sound mind? A. Certainly. — Q. During your visits to this lady did you discover there was any presence of liquor? A. At the last visit she was taking her dinner—I presume so—it was about six o'clock. — Q. Your visit on the 6th of January was expressly to ascertain whether she was of sound mind or not? A. Yes. — Q. And did you conceive the inquiries about her property you made, and the answers she gave, were sufficient to enable you to judge? A. Yes. — Q. Suppose she said she had sold property to the Water Works property, and gave no account of what she had done with that property, what would you say then—would you not have gone on further to say, what did you do with it? A. I should not ask her what she did with it, as I should presume she received it if she had sold it. — Q. Would you not inquire what she had done with it? A. I should not consider that a test of the soundness of her mind.

*George Simpson*, sworn, and examined by Mr. Symons.—Q. Are you a member of the Royal College of Surgeons in England? A. I am a fellow of the College of Surgeons. — Q. Have you been in practice as a surgeon during twenty-seven years? A. I have. I passed in the year 1824. — Q. Have you been engaged as a lecturer on anatomy? A. I have, some years since. — Q. Are you the surgeon to the National Vaccine Establishment and the Westminster General Dispensary? A. I was for several years surgeon to the Westminster General Dispensary, and I am now surgeon to the National Vaccine Establishment, a government appointment. — Q. Do you recollect seeing Mrs. Cumming at York House, Battersea, in 1846. A. Yes. — Q. What time? A. On the 16th September in that year, in September, 1846. — Q. Did you see her with a view of examining her state of mind? A. Yes.

*Witness.*—I think it right to explain that I was requested to attend at the Horns Tavern to watch the proceedings, and to visit her professionally when the case was adjourned at York House Asylum.

Q. You attended her professionally from the 16th September to the close of the commission? A. Yes. — Q. And attended the commission at the Horns Tavern? A. I did so. — Q. Did you have conversations with her upon the occasions of your first visit? A. I had. — Q. Do you recollect the substance of those conversations? A. I told her, on being introduced to her, that I had been requested to attend her to examine into the state of her health. At first I did not immediately allude to any particular subject, but entered into a general conversation as to the asylum she was confined in, the number of patients that were there, and as to whether she was under any medical treatment. — Q. What were her answers to those inquiries? A. The answers that I should have expected—there was nothing particular occurred. — Q. But they were the answers which you would have expected from a perfectly rational mind? A. Yes. I then asked her how long she had been in the asylum. She said she had been removed there in the month of May, I think. I do not recollect the date, but she said the month of May. — Q. In that year? A. In that year. — Q. Will you proceed, if you please? A. She complained of the position in which she was then placed, and said that her family had placed her there. I asked her who she meant by her family? And she said her daughters and their husbands. I think she said that her house had been seized. She first said that her liberty had been taken from her; that her house, papers and property, I think, had been seized, and that she was without friends or money. — Q. Did she describe to you the mode of her removal to the asylum? A. No, she did not. — Q. This was a general observation of hers? A. This was an observation as to how she came there. She said that her friends had been denied to her. I think she mentioned the name of Farrer at the time that her friends had been denied access to her. She said, "I have been associated with those people," pointing to two females who were mad, who were walking in the grounds at the time. After this conversation, I thought it right to touch on the points which I had heard before the learned Commissioner, and I asked her as to her dislike to her children, and why she had taken that dislike to her children. She said "that her second daughter had married contrary to her wishes, and that her other daughter, Mrs. Ince, had encouraged it." I think she said she either was married from her house, or that she went to church with her. She became a good deal excited at that moment, and appealed to me. She said, "Sir, I do not know whether you have daughters of your own, but do you not think I have sufficient grounds of resentment? Would you not have acted in a similar manner?" — Q. Did she refer to her husband at all? A. I think she said her husband was an invalid at that time. I believe her husband was dead, but she was not aware of the fact, nor was I aware of it till this present inquiry. I have forgotten the circumstance. — Q. Her husband was dead, and she was not aware of the death? A. She did not say her husband was dead. — Q. Will you proceed if you please? A. She said that none of her family had been there to make inquiries after her. I then referred to the infidelity of her husband, which I understood she had mentioned. I believe I used the word delusion to her, and she said it was no delusion, that it was a fact, as she could prove by witnesses. I now recollect that she did mention her husband, because she brought up the charge by saying that she could prove it by the nurses who attended upon him, for that he put his hands down their bosoms. — Q. Will you proceed? A. I said I understood she had made use of very bad language, and she admitted the fact. She said that she was of an irritable, to use her own term, temper; and she added it was done under great provocation, but she was sorry for having done so. — Q. That was the language she had used? A. Yes; she had spoken of her husband being a very irritable man, which led to my putting some questions to her respecting a hot poker which I had heard mentioned at the former inquiry. I was induced to put those questions, having made minutes of them at the time.—Q. You referred to the use of a hot poker? A. She said it was perfectly true that he had made an attack upon her, and that the police were called in, and that they would be produced before the Commissioner. — Q. That he had made an attack upon her with a hot poker, did she say? A. Yes; I think those were her words. I remained about an hour and three quarters with her on each occasion. — Q. Did you refer to any other delusion at that time? A. I do not recollect anything particular that passed regarding that. — Q. You remained an hour and three quarters? A. At each visit, I think. — Q. Had you conversations on other subjects than this to which you referred? A. Yes; there was a general conversation. I did not dwell particularly, but previously to leaving, I thought it necessary to put these questions. — Q. Upon the occasion of that first visit, did you form any opinion as to the state of her mind? A. Yes; I thought from what I had heard that I should wish to visit her again, because I am quite aware on visiting patients, it is sometimes impossible at one interview to arrive at any conclusion. — Q. Before coming to any decided opinion you had a wish to see her again? A. I did. I told her I should see her in two or three days. — Q. Do you recollect whether this first interview you had was during a period of some days over which the commission had adjourned? This was after the commission had been adjourned in the interval you saw her again? A. I saw her on the 18th, two days afterwards. — Q. Will you state what occurred on that interview? A. She received me as before. She was very agreeable and ladylike. I think I told her I had omitted in my first inquiry, to speak with respect to her fondness for dumb animals, alluding to the cats and pigeons. She said it was perfectly true that she was fond of dumb animals, cats, and that she also kept pigeons. But I said, I am informed that you kept them shut up in the room and the windows closed. And she said, "why, I had just removed to a new residence, and I was afraid my pigeons would fly away, and that was why I kept the windows closed." I do not know whether she had removed or not, but that was her reply. She said, "is there anything extraordinary in my keeping cats or pigeons?" I said, "certainly I did not think so." I then tested her numerical powers by setting her two sums. I thought it was in division, but I find from minutes on each interview of what passed, and I find they were two sums in addition. — Q. Did you see anything that occurred on the part of Mrs. Cumming during the progress of the commission, to change the opinion you had formed and expressed in your report? A. Nothing; on the contrary to confirm it.

Mr. James.—Have you any objection to take your report on oath, of what you saw, to the conclusions on which you arrive? A. No. I made minutes of each interview, and drew my report immediately. — Q. On the third day of your seeing her, that was the 15th, I believe? A. The third day. I cannot speak correctly as to the date. It was the Saturday following the 18th. — Q. On that occasion did she manifest some irritability of temper when you saw her. A. She did. I was shown up stairs in the drawing-room, and she came up very much agitated and excited. I asked her the cause of it, and I think she said, that she first saw me crossing the garden or grounds, and the door which was on the right being open she saw me go by, and observed to the matron that she was wanted; that the matron said it was not the case, but that it was a gentleman for some other patient; she said that she was fearful that the medical gentlemen who were about visiting her were about to be denied, as her friends previously had been, which was the cause of her being so much excited. She was very much excited, and trembled a great deal upon the occasion. — Q. Under these circumstances, did the excitement in which you found her, strike you as being unnatural? A. No. — Q. Did she argue upon the matter, and give you a reason? A. She said that her friends had previously been denied access to her, and that she was fearful the same thing was about being adopted. — Q. Did you upon that occasion allude to her alleged uncleanly habits? A. I did so. — Q. What answer or explanation did she give you? A. She denied them in strong terms, in fact, almost refused to answer them. I told her that I had heard it from a gentleman who was at the head of the asylum, whom I previously knew, and who, I ought to have stated, stopped me on my second visit going down stairs; he met me with his case-book in his hand; it was Dr. Millengen, and he called me aside. I repeated to her at the third interview what Dr. Millengen had said as regarded a white cambric pocket handkerchief; she denied it most solemnly, and said she had every convenience she could wish for, and was it likely that she should have indulged in such filthy habits. — Q. Did you see her afterwards in the asylum? A. Not after that. — Q. You saw her at the Horns' Tavern? A. I did. — Q. Now, I will ask you again, the result of these interviews in your mind as to her state of mind? A. I considered her in a perfectly sound state of mind after the explanations she had given me on the matters which I considered it my duty to touch upon. — Q. Did you see her again on the 27th December, 1851? A. I did; but I saw nothing of Mrs. Cumming, or of her solicitor, or of any of her family whatever, until about a fortnight since. — Q. You had not seen her in the interval at all? A. No. — Q. Nor any of the parties connected with her? A. None whatever. — Q. How long were you with her on that occasion? A. About three-quarters of an hour, I should think, to an hour. — Q. What conversation had you with her on your first introduction? — A. I said I was very sorry to be obliged to visit her on the unpleasant affair of the previous inquiry, and I believe I said, "I am glad to see you look so well, ma'am," and she said, "Well, you do not know what I have gone through." I think that was her expression. I must state to you that at this interview Mr. Haynes was present. Mr. Haynes went with me, and introduced me. — Q. What conversation had you with her? A. On Mr. Haynes introducing me, he said, "Do you recollect this gentleman?" and she stopped for a few moments and did not recollect me, and he said, "This is Mr. Simpson," and she said, "Oh

I remember, you visited me at York House Asylum." — Q. Will you proceed? A. She stated that she had gone through a great deal during the six years—that her family had not left her alone. She said she had been persecuted by them; that she had two chancery suits brought against her, and had been indicted for perjury, and had been brought from her residence in Brighton, by railroad, to which she said she had a great objection. "In fact," she says, "I am now considered a lunatic, for that female who showed you upstairs is my keeper. That is a nurse," I think she said, "appointed by the Lord Chancellor." There was a general conversation kept up. I asked her as to her state of health—what it had been. I remarked that one of her eyes was very much inflamed—that she laboured under chronic ophthalmia; and I think she said it was of some standing; and I was about writing a prescription, but she said Dr. Caldwell was in attendance upon her, and of course I did not interfere; and I said, "I remember our having a long conversation respecting the cats and pigeons," and she laughed, and said, "Yes; I remember it very well." — Q. Did you allude to the previous conversation at York House? A. Yes, I was alluding to that. — Q. Did she laugh? A. She laughed. She complained bitterly of having been persecuted; that seemed to be her principal cause of complaint to me. On leaving her, I explained to Mr. Haynes that on my next interview I should wish to see her alone, and he said, "Certainly." — Q. Was there any other conversation on this occasion on general subjects that you have any recollection of? A. Not that I can recollect. I think she spoke of her daughter on this occasion. She still entertained the same feeling as regarded her children; and I ought to have mentioned, that in the former inquiry she particularly alluded to the marriage of her daughter with a soldier. That was in the first inquiry, and the same feeling seemed to exist in her mind as regarded her family. — Q. Did you attend her upon the occasion when an appointment was made for your seeing her again? A. I did. — Q. When was that? A. On Monday, the 29th. — Q. Of December? A. Of December, 1851. — Q. What time of the day did you visit her on that occasion? A. I had made an appointment for one o'clock with the footman or groom in attendance, and when I arrived, I found two other medical gentlemen were in attendance upon her, Dr. Diamond and Mr. Davey. — Q. Did you wait until those gentlemen had left her? A. I waited about an hour, and rang the bell for the servant, saying my appointment was for one o'clock, and that I had been in attendance an hour, and to know whether they would be there long, and I think they left almost immediately afterwards. — Q. Did you upon that proceed to Mrs. Cumming's room? A. I did. — Q. Was anybody in the room with you? A. There was a lady sitting there, who I have since ascertained is a Mrs. Moore, who came out of court yesterday; she said she was the widow, at the time, of a medical man. — Q. In what state did you find her when you got into the room? A. I was very much surprised to find her so perfectly calm and collected, and in good humour, for she laughed on entering, and said, "Well, upon my word, I think you doctors will drive me mad." I think those were her words. — Q. Did she recognise you? A. Yes. She begged I would be seated; and she was either at lunch or dinner. There was some rumpsteak, I think, on the table, and I begged her to proceed with her dinner; and she said, "No, she had quite dinner enough; that the other gentlemen wished her to go on with her dinner, but that she could not eat anything more." Our conversation principally was upon the questions she related to me of what they had asked her. She said, Dr. Diamond she knew before, but the other gentleman she did not, Dr. Davey. She said that doctor had put questions to her, and wished her to recollect the dates when she was taken to the asylum, and she said, "I knew very well that those gentlemen would bring everything against me, and I said to them, 'I would not be fixed to a date, but I will answer it there, when in the presence of the judge and the jury, as the inquiry will soon take place.'" That Dr. Diamond was very anxious that she should see her daughter, Mrs. Ince, and asked her if she would see her; that Mrs. Ince was very much concerned about her, and would like to see her; and she said, "Certainly not." She said Dr. Diamond remarked to her, "I do not think you seem well to-day, Mrs. Cumming, for you are not so communicative on this occasion as when I last saw you." — Q. Did she say what her answers had been? A. She said, "Yes, I am quite well;" but she said, "I was aware that they were sent by my family, and I wished to be particular and upright in everything I said." — Q. Did you speak to her on this occasion with reference to her alleged delusions? A. I do not think I did. I think it was on the former inquiry. I cannot recollect whether I did at this time. The conversation I had with her was principally her telling me what had transpired between those medical gentlemen and herself. — Q. Did you, on the first occasion at the Queen's Road, have conversation with her with reference to her alleged delusions? A. I did. — Q. Did you speak to her about the allegation that she had been endeavoured to be poisoned by her children? A. No, she never mentioned that circumstance to me from the commencement, and never once alluded to it. She spoke of persecution, and her aversion to her children. I think she said they had hunted her from place to place; and on one occasion, I cannot say which, she said, "It is my money they want, and if they would only wait sufficiently long, they should have it;" or something to that purpose, if they would only wait a sufficient time. — Q. What was the length of the conversation you had with her on this second interview? A. Three-quarters of an hour, it might be an hour. She stated that those medical gentlemen repeatedly said, "Why, Mrs. Cumming we cannot make anything of you to-day; as it appears clearly to us, that you would rather see our backs than our faces;" to which she replied, "It would be very unladylike in me to express such an opinion, whatever I may think." Upon which, she said, they left the room. — Q. On the occasion of either of those interviews was there any reference made to her entertaining the idea that her daughters had endeavoured to strangle her? A. None whatever. — Q. Did you not refer to it yourself, nor did she? A. I did not, nor at the asylum either. — Q. Have you seen her since then? A. I saw her in court the other day twice. — Q. Have you observed any change in her person since you saw her in 1846? A. I was not aware that she was so helpless as I saw her when she was brought into court. When I saw her in 1846, she walked up and down stairs the same as any other person. — Q. From the opportunities you have had of forming an opinion of the state of her mind, are you of opinion that she is of sound mind? A. Certainly. — Q. Perfectly sound? A. I consider her of perfectly sound mind. — Q. Have you seen anything in her to indicate the existence of any delusion at all? A. I think without explanation they might be called delusions; but I think from what she has stated, and what I have since ascertained of the facts, I do not consider them delusions.

Cross-examined by Sir F. Thesiger.—Q. Without explanations, you say they might be considered delusions? A. Without explanation. — Q. Did you not desire to see Mrs. Cumming without the presence of Mrs. Moore? A. She was sitting in the room. I was not aware she was there till I was shown in; and I think I said at the time, "Who is this lady?" and I do not know whether it was Mrs. Cumming or the lady herself who said, "I am a friend of Mrs. Cumming, and I have permission to visit her." — Q. But you did not ask anything about her daughter having attempted to murder her, or poison her, or strangle her. A. No, I did not. — Q. Did you on any occasion? A. Never; for I was not aware of the fact. Q. Did you ask her anything upon the subject of the milk being poisoned? A. Oh, yes, I remember that very well. — Q. On which of the occasions? A. I think it was on the first occasion. — Q. When Mr. Haynes was there? A. Yes; she said that her fowls, I think, not pigeons—that her fowls had been poisoned—that there had been poison put. — Q. Did she say where? A. No, she hinted. She said something that an attempt had been made to poison her—that her fowls had died. — Q. That an attempt had been made to poison—that her fowls had died? A. Yes. — Q. You did not mention that? A. No; your mentioning the circumstances reminded me of it. — Q. You remember that? A. Yes, that was the first time. — Q. How came she to mention it? Did you put in a question to her about it? A. No; I was asking her how her general health had been, and relating circumstances connected with her family, and she said, "I have been attempted to be poisoned, as I can prove," I think she said; and I think she mentioned Dr. Barnes's name: she did, for she said it was analyzed, and poison was ascertained to exist. I remember her particularly stating that. — Q. That you had forgotten entirely before? A. I did not know that it had been analyzed, or any thing concerning it. — Q. I want to know whether you agree that it has been observed by every medical person who has the care of lunatics—"do they sometimes acquire the habit of concealing their impressions, particularly if frequently questioned respecting them,"

and that it requires some art and address to bring them to the subject without putting them on their guard? A. I do agree in that opinion. I think I state so in my report. — Q. I understood you that, in the preceding inquiry in 1846, it had been going on for some time before you were called in at all. A. It might, perhaps, have been going on. I was present on two or three days, and then I was requested to visit her at the asylum during the adjournment. — Q. Was Mrs. Cumming present during those three days? A. I think every day I was there; and I was really astonished at the manner in which she conducted herself. — Q. She heard the different points that were alleged? A. She did, and I made dates and memorandums of circumstances that had occurred, and examined her on those at the asylum. — Q. She knew the grounds which were alleged to establish her insanity or unsoundness of mind? A. She did. — Q. And she knew perfectly well that the inquiry was going on as to her state of mind? A. She did.

Re-examined by Mr. James.—I do not know whether you heard the medical evidence on the other side? A. I have. — Q. Has there been from their evidence any concealment by the lady of her delusions.

Sir Frederick Thesiger.—I do not know that my friend is at liberty to ask him to form an opinion upon the evidence which has been given.

Mr. James.—No, you are right. — Q. Have you, on her part, noticed any studied concealment of any delusion? A. Never. I have always found her most ready to give any answer. — Q. You have been asked if you arrive at conclusions without an explanation of the facts. If a person told you there had been an attempt made to poison her, and alluded to the fact of an analysis being made, should you not inquire into those facts before you arrived at conclusion whether it was a delusion or not? A. Most certainly. — Q. Now, although my learned friend cited from some book——

Sir Frederick Thesiger.—Dr. Pritchard.

Mr. James.—That insane persons can and do frequently conceal their delusions, is it not also a fact of disease of the mind, that where delusions exist they are constantly haunting the mind, and constantly a subject of conversation? A. Yes, constantly. — Q. I believe in 1846, a delusion, or one of the delusions alleged, was perversion of affection towards her children, and one of the main grounds? A. It was so. — Q. We know now that Dr. Barnes and the poison occurred subsequently to 1846. A. I am aware of that. — Q. Was there any allusion at all of any attempt to poison her before these facts occurred? A. There never was any allusion made to it.

Mr. James.—I find that a question arose yesterday with reference to the identity of Captain Cumming, as the party upon whom the order of affiliation was made, and his notice was put in, and I now propose to prove that the signature to the notice is in the handwriting of Captain Cumming.

*Mr. Farrar* re-called.

By Mr. James.—To the best of your belief is that Captain Cumming's handwriting? (handing to the witness the notice of appeal.) A. It is.

Mr. James.—It is a notice of appeal, dated the 4th of October, 1822, against the order of affiliation.

A Juryman.—That is thirty-five years ago?

Mr. James.—Yes, sir.

A Juryman.—That we have nothing to do with.

Mr. James.—Pardon me, sir; some gentlemen of the jury may consider it material, and some may not. When we produced this paper yesterday, there was an objection raised by my friend, Sir Frederick Thesiger, that there was no evidence of identity—it is for the jury, by-and-by, to say whether this evidence is relevant or not. It appeared to us, as the objection was made, to prove that Captain Cumming was really the party named.

*Henry Swan Caldwell, Esq., M.D.*, examined by Mr. James.—Q. I believe you reside at North Addington-place, Camberwell? A. Yes. — Q. I believe you are not a member of the College of Physicians? A. Not of the London college. — Q. Have you graduated at any foreign university? A. I graduated at the University of Paris and of Glasgow. — Q. Have you been in practice for many years? A. I have been twenty-one years in Camberwell and practised previously at Paris. — Q. When were you called in on the first occasion to see Mrs. Cumming? A. On the 12th of May, 1847. — Q. Where did you then see her? A. At Mrs. Hutchison's, at Vauxhall. — Q. Was she then on a visit at Mrs. Hutchison's, or residing there? A. On a visit there. — Q. What did you attend her for? A. For an affection of the stomach. — Q. How long did you attend her then? A. I attended her till the 4th of June. — Q. Did you see her frequently at that period? A. I saw her every day. — Q. Did you know at that time, when you were first called in, that she was the lady whose mind had been the subject of inquiry and the commission of 1846? A. I did not till after a few days. — Q. I need scarcely ask you, if you directed your attention more particularly than you otherwise might have done to her state of mind and conduct generally? A. She appeared then, very unwell and rather depressed. — Q. Was it the fact that, from having heard that her mind had been the subject of investigation, your attention was more directed to her position and conduct than to any other? A. Yes, it was. — Q. Did anything strike you—I will not go into detail here—did anything strike you as strange or remarkable about her conduct at that time? A. Not in her conduct; nothing in her conduct. — Q. In her manner was there anything; what sort of person did you find her to be? A. She seemed rather depressed in spirit as well as infirm in her body. — Q. When did you again attend her? A. I attended her at Camberwell, Clifton-place, Camberwell, from the 4th June to July 16th. — Q. What for, then? A. She had not perfectly recovered of her complaint for which I attended her at Vauxhall. — Q. Did you attend her every day? A. Mostly every day, and sometimes every other day. — Q. When again did you attend her? A. At St. John's Wood, from August 1st to December 17th. — Q. What year; the same year 1847? A. The same year. — Q. What was she suffering from then? A. She had various complaints—an inflamed eye, which she had suffered from for a long time, and defective appetite. — Q. Was that at the Queen's-road, St. John's Wood? A. Yes. — Q. When did you attend her again, that carries it up to December, 1847? A. From January 3rd to February 28th, 1848. — Q. When again? A. From March 27th to August 21st, 1849.

A Juryman.—Q. Just previous to her going to Wales? A. The next day she went to Wales.

Mr. James.—Q. When did you attend her again? A. At Cheltenham, March 22nd and 23rd. — Q. Was she *en route* then from Wales to London? A. Yes. — Q. When again? A. At St. John's Wood, April 16th. — Q. Was that at the Queen's-road, where she is now? A. Yes; from April 16th to January 2nd, 1851. — Q. Does that embrace any part of the periods of Eleanor Hickey and Mary Rainey being the servants there? A. I believe they were there. I do not remember Hickey, but I remember Mary Rainey being there. — Q. You remember her very well? A. Yes. — Q. Do you remember the woman Hickey? A. I scarcely remember her, but I remember the children that appeared here. — Q. That is Mary Ann Hickey and Ellen Thompson? A. I remember having seen children there, without knowing who they were. — Q. But they are the same children? A. The same children. — Q. While Mary Rainey was there, were you in the habit constantly of visiting Mrs. Cumming? A. Very frequently; three or four times a week sometimes. — Q. Do you remember the partiality of Mrs. Cumming for her cats? A. Yes, I do. — Q. Were you in the habit two or three times a week of being in her bed-room? A. Yes. — Q. In what state did you find her bed-room? A. It was sometimes close when I went into it from the fresh air. I found the difference as in other bed-rooms. — Q. Was she suffering at that time from inflammation of the eye? A. Yes; and a rheumatic affection. — Q. In what state was the room as to cleanliness from these cats—we have had a description of that? A. It was pretty well. — Q. Was there anything so filthy about it? A. I never saw any filth in Mrs. Cumming's house but once, and that was immediately taken away by the servant. — Q. You mean from cats? A. From cats. — Q. Where was it you saw that? A. That was in the Edgware-road. — Q. What room was that in? A. That was in the sitting-room. — Q. And that was immediately taken away? A. That was immediately taken away. — Q. By whose orders? A. Mrs. Cumming pointed the servant to it, and it was immediately taken away. — Q. Did you see in that room while you were attending her, during the time Mary Rainey was there, heaps of dirt under the bed, or did you smell it?

A. I did not look under the bed. — Q. But you could detect the presence of such filth as that if it was there, could you not? A. I do not imagine that such filth was to be found there. — Q. She left, if I remember rightly, somewhere about the end of January?

Sir. F. Thesiger.—The beginning of February.

Mr. James.—Did you attend to nearly the end of January? A. Herbert Villa comes next. — Q. When did you next attend her? A. January 3rd to February 9th. — Q. We have got to the 2nd January, 1851. Did you resume your attendance, or was there any break? A. No break there. — Q. And how far did your continuous attendance go? You have told us the 2nd of January. Was that broken off then, or did you follow her to her residence? A. I recollect very well that I went to Herbert Villa, expecting, from what she said to me the previous time, that she would be there that day, and I found the coachman waiting there to conduct me to her other residence, that she had not had time to remove. — Q. How far into the year 1851 did you attend her continuously? A. Stamford-street comes next, from February 11th to March 10th. — Q. What is the time of the Edgware-road? A. March the 10th to June 9th.

Mr. James. — Do you go on beyond the 9th of June? A. At Worthing, August the 6th. — Q. Well, were you examined before the Commissioners of Lunacy? A. Yes. — Q. When did you see her again? A. At Effra Hall. — Q. After October that would be? A. On the 9th I went, but they would not allow me to see her. — Q. When did you see her? A. 17th November. — Q. You went to see her, but they refused to allow you? A. On the 9th they did, but on the 17th I saw her. — Q. And when again? A. St. John's Wood, November 12th to January the 1st. — Q. Did you hear the evidence of Mary Rainey? A. Yes, I did. — Q. And of the children as to the dirt and filth? A. I did. — Q. As far as you heard that statement, from what you stated, was that true, or is it exaggerated? A. I thought it very much exaggerated. — Q. Was she labouring under disease, that rendered it impossible that she could prevent a great deal of what they alleged happening? A. Yes; I have spoken to her about threats, from the description given by Mary Rainey, and she alluded to her own infirmity. — Q. Do you believe that many of the things which have been stated occurred involuntarily on her part? A. I do. — Q. You heard their evidence? A. I heard their evidence. — Q. And you say here, on your oath, a great deal of that is exaggerated? A. It is. — Q. I ask you now, do you know, from attending her as a doctor, that she was in that state of bodily infirmity, that filth of this kind would occur involuntarily on her part? A. It was for that very complaint I was attending her. — Q. From what you have seen of her so constantly as you have, and the opinion you have formed, is she, in your opinion, of sound or unsound mind? A. Of sound mind. — Q. Have you heard her express herself strongly about her daughters; her children? A. Frequently. — Q. Without going into the detail of every conversation, in what way generally has she spoken of her children, and their conduct towards her? A. They had used her very ill, by having her confined to a madhouse, and not visiting her there while she was in confinement. — Q. Did she speak to you of the arrest for perjury, that was attempted upon her when she was at Stamford-street? A. Yes, when we gave the certificate that she was not fit to be removed under that arrest. — Q. Did you give a certificate that she was not fit to be removed under that arrest? A. Yes; conjointly with Mr. Johnson, a surgeon, in the Waterloo-road; I saw the bed-room door had been forced. — Q. Was she at that time in a fit state to be taken to a police-office? A. Not at all. — Q. Have you heard her speak about her children? Have you heard her mention the subject of the poisoning and the analysis? A. I never heard about the poisoning all the time I was with her; she never mentioned that to me. — What has she chiefly mentioned about her daughters' conduct? A. What I have just stated. — Q. Where have the cats been generally? A. Sometimes in the bed-room, sometimes on the stairs, sometimes in the garden. — Q. How many have you seen there? A. I once saw four and a kitten. — Q. Did you see anything so extravagant or eccentric in or about the attachment towards them, that struck you as a test of insanity? A. No; I considered it was matter of choice. — Q. She was left very much alone there when she was ill, was she not? A. Very much. — Q. You say you attended before the Commissioners of Lunacy, and was examined? A. Yes. — Q. I

believe a complaint had been made to the Commissioners of Lunacy that Mrs. Cumming was under duresse; that she was a person under restraint, and wanted force to go where she pleased? A. Yes. — Q. I believe you gave your evidence to show she was, in fact, able to go where she pleased? A. Yes; I gave my evidence according to all the circumstances I knew about her. — Q. And that, possibly, you know led to a report which has been read here, to say that the Commissioners refused to interfere because they found she was a free agent? A. Yes.

Cross-examined by Sir F. Thesiger.—On the 6th of August I think you visited her at Worthing? A. I did. — Q. Was not there an application to you from this gentleman (pointing to Mr. Turner's clerk) on the 16th of August, and did you not refuse to give any information where she was? A. The first time he called; he called twice. I do not know about the second time. I asked him who he was; whether he was an officer or not. — Q. Did you refuse to tell him where she was? A. Yes, because I thought it very rude for a professional man to behave to another professional man in that way. — Q. You refused to tell him? A. I refused to tell him, and requested him to tell Mr. Turner never to send again.

[Dr. Caldwell was subjected to a long cross-examination, relative to some acceptances he had received from Mrs. Cumming in payment of his bills for professional attendance. We give this *resumé* of a series of questions put to Dr. Caldwell, which had direct bearing upon the question at issue.]

Re-examined by Mr. James.—Did you know at that time that they intended to take her into custody, at Stamford-street, on a charge of perjury? A. I did. — Q. Who was the gentleman who applied to you, is he in the room? A. That is the person (pointing to Mr. Turner's clerk); I asked him who he was, and he said he was an Irishman. — Q. This person called upon you and recommended himself in the first instance, saying, he was an Irishman? A. Yes, and that if I would call on Mr. Turner, at No. 9, Carey-street, Lincoln's-inn, he would tell me all about it. — Q. You are attending her professionally, and do you consider that she is in a precarious state of health? A. She is. — Q. Do you know whether it is the truth, that she does not take the physic you prescribe for her? A. I think there is some truth in that; but not all the truth. I have generally presented it to her myself when I have found that to be the case, and she has taken it. — Q. Have you reason to believe that she takes, to use your own words, either wine or brandy and water, on what is called "the sly?" A. I think she takes each occasionally. I have recommended it to her sometimes, when I have thought it necessary. — Q. You say you got inconsistent accounts from the servants? A. Some have told me that she does take it, and others tell me she does not take it.

Examined by the Jury.—During your visits to this lady, have you had to treat her for delirium tremens? A. No. — Q. And would you consider it necessary she should have a certain quantity of brandy and wine a day? A. Not every day; when occasions require it.

*W. H. Hodding, Esq.* examined by Mr. James.—Are you a general practitioner in Gloucester-place, Portman-square? A. I am.—Q. How long have you been in practice? A. Twenty-six years. — Q. Do you know Messrs. Birch and Davis, Solicitors, of Newport? A. I do not know Mr. Birch at all; I know Mr. Davis. — Q. Of the same firm? A. Of the same firm. — Q. Do you remember being applied to in May, 1851, to see Mrs. Cumming, with reference to the conveyance of some property to Sir Charles Morgan? A. I do. — Q. At whose request did you see her? A. At Mr. Davis's request. — Q. Upon what object were you to see her? A. To satisfy Mr. Davis that she was sufficiently sound to execute a deed of sale. — Q. A conveyance to Sir Charles Morgan? A. Yes. — Q. Were they the solicitors at that time of Sir Charles Morgan in the purchase? A. Yes.— Q. Where did you see her? A. In the Edgware-road. — Q. At her house? A. No; in apartments there. — Q. Have you got the exact day you saw her? A. No. — Q. What month was it? A. In May, 1851. — Q. What passed when you saw her; what did you say to her to form your judgment? A. I asked her several questions relative to the nature of the property she was about to transfer, and of what property she was possessed, and a great many questions which I considered necessary, and I left her under the impression she was a very competent person to execute a deed; in fact, a very clever person.— Q. You believe she is a person of cunning, active mind? A. Yes. — Q. Did you leave her under the impression she was competent to execute the deed, and did you complete the purchase? A. Yes. — Q. You never saw her before, I believe? A. I never saw her before, or heard of her. — Q. How long were you with her? A. I should think half an hour. — Q. Have you heard that her mind had been a subject of inquiry in a previous commission? A. I did not hear there had been a previous commission; but I heard from Mr. Davis, there was some doubt as to her mind being perfectly sound. — Q. And that directed your attention particularly to her? A. Exactly. — Q. Have you seen her since? A. I have. — Q. How often have you seen her since? A. I have seen her three times very recently. — Q. Will you give to the Jury the dates when you saw her? A. I think it was the day after Christmas Day of last year. — Q. The 25th of December? A. Yes, about the 30th and 31st, I should think. — Q. I believe you not only reported she was competent to sign the deed, but you attested the deed yourself? A. I did. — Q. Was that deed read over to her? A. Yes. — Q. Did she seem to understand the purport of it? A. Perfectly. — Q. Or you would not have attested it? A. I would not have attested it. — Q. Was the money paid to her? A. The money was handed to her in my presence, and she was about to go, and then I left the room; I thought my business was done. — Q. Did you leave with the belief that, having attested the deed, she thoroughly understood what passed? A. I did. — Q. In your three last interviews with her in December, last year, had you any reason, from your own interview, to alter your opinion? A. Not the least. — Q. Did you talk to her at all about her daughters? A. I spoke to her about this coming commission, and that naturally led to conversation about her daughters; she merely said they had treated her very ill, and I did not think it necessary to pursue the conversation any further. — Q. Is it your opinion that she is a person of sound mind? A. It is quite so.

Cross-examined by Sir Frederick Thesiger.—Who did you see in London before you went to Mrs. Cumming in the Edgware-road? A. I was taken by Mr. Davis; he came to my house and fetched me. — Q. Was Mr. Haynes present during the time you mentioned at Mrs. Cumming's? A. He was. — Q. Was anybody else present during that half-hour's examination? A. There was Mr. Davis, myself, and Mr. Haynes, and a gentleman who I understood was a solicitor, but whose name I do not know. — Q. During the half-hour's conversation with her? A. Yes. — Q. Was the deed executed then at the same time after that conversation? A. That was the only time I saw Mrs. Cumming. — Q. You went there with Mr. Haynes? A. No, I did not. — Q. I thought Mr. Haynes had sent to you? A. No; Birch and Davis. — Q. You did not go with Mr. Haynes, but found him there? A. I found him there; and I believe he objected to my being there, as being unnecessary. — Q. You left her counting the money? A. She was just about to do it. — Q. Who did you leave with her? A. Mr. Davis, Mr. Haynes, and this other gentleman, whose name I do not know. — Q. I understand in your three subsequent interviews she began to speak of her daughters behaving ill upon the subject of the present commission, and you did not pursue the inquiry further? A. Exactly.

Mrs. *Elizabeth Davis*, sworn. Examined by Mr. James.—Where do you reside? A. At 27, Deane-street, Commercial-road East. — Q. Were you at one time, in May, 1846, matron of the York House Asylum? A. I was. — Q. Where Mrs. Cumming was confined? A. I was. — Q. That was kept at the time by Dr. Millengen? A. It was. — Q. Did you read in the newspapers on Saturday last this investigation that was going on? A. I did yesterday. — Q. In consequence of reading the inquiry in the "Times" newspaper, what steps did you take? did you go to find out the counsel or the attorneys? A. I commissioned my son to do so. — Q. Do you remember Mrs. Cumming coming to the York House Asylum? A. I do. — Q. How was she brought there? A. By two nurses. — Q. Do you remember whether she had any strait-waistcoat put upon her? A. Yes. — Q. Were you there when she was brought to the house? A. I was there when she was brought in the house, but I did not see her till she was brought in the parlour.— Q. When you saw her, had she a strait-waistcoat on her? A. No; but she informed me she had had. — Q. What did Mrs. Cumming tell you when you first saw her in the parlour of the York House Asylum? A. That she had been very unkindly treated; roughly treated by the nurses who took her. — Q. And did she tell you anything about a strait-waistcoat? A. Yes, that she had had the strait-waistcoat put upon her. — Q. I believe you left the asylum early in August, before the commission? A. I did. — Q. From the time that she was brought in May up to August, a period of about four months? A. I left before August. — Q. When did you leave? A. In July. — Q. Was she there about five months? A. I think about that time, I cannot say to a day or a week. — Q. Did you attend upon her as matron? A. I was the ladies' superintendent for the comfort of the house, not in any particular position. — Q. Had you many conversations with her during the time you were there? A. I was with her daily, and at every meal.— Q. Did you ever see any violence upon her part which would require a strait-waistcoat to be put upon her? A. Oh, dear no. — Q. What was her conduct, so far as you observed it while she was there? A. That of a highly respectable lady. She conducted herself with great propriety, I considered. — Q. Did you form any impression as to whether she was of sound or unsound mind? — A. I think she was of stronger mind than I am myself. — Q. Did you form your impression from her conduct? A. Yes; considering her years, I think she was of very sound understanding. — Q. Did she complain to you of the conduct of her daughters? A. She lamented their cruel treatment to her in tears many times. — Q. While she was there, did any of the daughters or her family come to see her, to your knowledge? A. I am not sure whether it was Mrs. Ince, but I think Mrs. Ince came on one instance. I think I saw her; I did not know whether it was Mrs. Ince or the other daughter, but it was one of them. — Q. Did either of them come upon more than one occasion? A. No; not that I know of. — Q. Do you remember whether the death of her husband was told while she was in the asylum, or not? A. I think it was communicated.—Q. By whom? A. By Dr. Millengen, I think. — Q. What were her habits as to cleanliness at the time she was there? A. She was more than cleanly, she was a nice lady. — Q. You saw her every day? A. Yes. — Q. At every meal? A. Yes. — Q. Did she take her meals with you? A. Yes. — Q. At what time did you form an impression in your mind as to whether she was of sound or unsound mind? A. It never occurred to me that she was still of unsound mind.

Cross-examined by Sir F. Thesiger.—What age did you consider her to be? A. I thought her between sixty and seventy years old when I knew her. — Q. Because you said considering her age, you do not think between sixty and seventy years of age an advanced age? A. It depends on constitution a good deal. — Q. You say Mrs. Ince or Mrs. Hooper, you do not know which, called once? A. Yes. — Q. Was Mrs. Cumming told of their calling? A. Yes. No, she was at the trial; she was afterwards. — Q. When she was told that her daughter had been, what did she say; did she express a wish to see her or not? A. No, she did not. — Q. Did she express a wish to see her? A. I believe she brought some articles of dress for her, but what they were I do not know.

Re-examined by Mr. James.—Do you remember whether she was told the daughter was there while she was there or not? A. No, she did not know of it. — Q. When she was brought there had she clothes? A. Scarcely anything — Q. How was she clothed during the four months she was there? A. After a time her wardrobe was sent. — Q. Do you know who sent it or who brought it? A. I think it was sent by the Inces' family, but what part I do not know. — Q. How long was she there without clothes, or comparatively without any clothes? A. Not a week, I should say. — Q. Where were you when the last commission was executed in 1846? You left before the commission? A. I did. I knew nothing of it till it was closed. — Q. Did you read that in the paper? A. No; I was informed of it by some relatives.

Mr. James.—Did you know the commission of 1846 had been executed? A. Not till after the business was terminated.

Examined by the Commissioner. — Did you tell Dr. Millengen your opinion of this lady's state of mind? A. No, I was never asked. — Q. But no person in their right mind ought to be in an asylum? A. But I could not control that. — Q. Were you a matron or superintendent? A. Yes. — Q. Did you express any doubt to Dr. Millengen about it? A. Dr. Millengen seemed so confirmed in his own opinion. — Q. Did you tell him your opinion? A. No.

*Forbes Winslow, Esq., M.D.*, sworn, and examined by Mr. James.—You reside in Albemarle-street, and have directed your attention very much to cases of insanity? A. I have. — Q. I believe you were selected by the Lords Justices to see Mrs. Cumming, and to report to their lordships your opinion of her state of mind? A. I was requested to do so by an order of the Lords Justices. — Q. When did you first see Mrs. Cumming in consequence of that official order? A. I would premise that I was first consulted about Mrs. Cumming on the 29th of October (1851). — Q. Last year? A. Last year. I was requested to go to the asylum at Effra Hall, where Mrs. Cumming was confined, for the purpose of ascertaining her actual condition of mind. I went on the 30th of October, accompanied by Dr. Barnes. On applying I was refused admittance. I understood that the matter was subsequently brought before the Lord Chancellor, and that, with the consent of counsel on both sides, it was arranged that I should have free access to the alleged lunatic. — Q. I believe you were named in the order were you not? A. On that occasion, I believe, there was no official order issued. I was informed that, with consent of counsel on both sides, it was agreed I should have free access to Mrs. Cumming for the purposes of my examination. On the day previously to my visiting her, Mr. Ince called upon me in Albemarle-street, and I had a conversation with him in relation to her case. On the 7th of November I went by myself to Effra Hall.

Mr. James.—Q. When did you first see her? A. On the 7th of November — Q. Were you alone with her, or with any other person? A. I was alone with her. Perhaps I may observe, that I met on that occasion at the asylum two of the Commissioners in Lunacy, who were paying Mrs. Cumming an official visit, and at their request I had a conversation with them in relation to the case. I felt an anxiety to make myself acquainted with all the facts of the case before my interview with her took place.—Q. You saw her officially? A. *Quasi* officially. I had an hour and a quarter's interview with her. — Q. Just state shortly to the Jury what passed? A. I observed that I had come to see her for the purpose of ascertaining her condition of mind and competency to manage herself and her property. I expressed a wish that she would communicate to me, without reserve, anything she had to say. She expressed a pleasure at seeing me; said that she presumed my visit was of a friendly character. In her unhappy situation, she was glad to see any one who at all appeared friendly disposed towards her. I asked her how long she had been at Effra Hall, and where she had come from? She replied, she was brought to London from Brighton to the asylum; that she had been there nearly a week. She then described to me what she alleged to be a very great outrage upon the liberty of her person; told me the police had broken into her private apartments at Brighton; that she had been subjected to several examinations; and that she had been forcibly dragged out of her house to the railway station, and brought up to London. She said that it was done by the advice and with the knowledge of her own family, of whose conduct she greatly complained. She observed, that it was not the first time that they had treated her in that brutal way. I said, are you not taking rather a harsh view of the conduct of your children? are there not other parties implicated in this matter? they may not be so bad as you appear to fancy them. She replied, that she was certain that her children were implicated in the transaction; that they had behaved, on various occasions, cruelly and unnaturally towards her; that they had made other attempts to deprive her of her property and her liberty. — Q. Did she allude, in the course of this conversation, to the previous commission in lunacy, and the proceedings in 1846? A. She referred to that circumstance in all the interviews I had with her. I am now merely generalizing what she said. I had an interview with her again upon the 8th November. She then went over the same ground in almost the same words. She complained of the irritation and anxiety to which she was subjected by being confined in an asylum, and of labouring under considerable bodily infirmity. She said that her health was declining; that she could obtain no sleep at night; that she had not slept for one hour during the time she had been in the asylum; prayed that I would intercede on her behalf, and obtain her release. She said, if she could be removed to her own residence, in St. John's Wood, she would be willing to submit to any number of examinations that were considered necessary for the purpose of satisfying the Lord Chancellor of her state of mind, and her competency to manage herself and her affairs. — Q. She said that? A. She said that she did not wish to shrink from any examination that might be considered necessary under the circumstances. She also remarked that prejudice would exist against her if she were allowed to remain in the asylum, and were to undergo the necessary number of examinations there. Perhaps I may be allowed to state, that I made, previously to her removal from the asylum, an affidavit in the case, expressing no opinion of mine as to her soundness or unsoundness of mind, but stating that I did not consider that she was in such a condition of mind as to justify her detention in a lunatic asylum.

Sir FREDERICK THESIGER.—That is not evidence.

*Dr. Winslow.*—I was merely stating that in the affidavit that has been referred to, I gave no opinion of Mrs. Cumming's soundness or unsoundness of mind.

Mr. JAMES.—Is it since then you formed an opinion? A. Yes. — Q. Upon that occasion did you make any inquiry of the nurse whether her statement as to not sleeping was correct? A. I did. — Q. Did the result of that confirm what Mrs. Cumming had told you? A. She confirmed Mrs. Cumming's statement. — Q. On what day did you see her? A. I paid her a series of visits after receiving the order of the Court of Appeal, extending from November 22nd to the day previously to this inquisition, a series of twenty visits in number, after Mrs. Cumming was removed from the asylum to her own residence, at St. John's Wood. I would premise, that I considered it my duty to make myself acquainted, as far as I could, with all the facts of the case, previously to my examination of Mrs. Cumming, with the view of entering upon its consideration in the full possession of all the particulars I could obtain with regard to it. The Lord Chancellor's Secretary of Lunacy forwarded to me a number of affidavits which had been made on both sides for my perusal; I had also interviews with Mr. Turner, the solicitor for the petitioners, with reference to Mrs. Cumming. I also saw the medical men who had been acquainted with her for a number of years, the servants, who knew her habits of life very intimately, and I also had a conversation with Mrs. Moore, who, I have understood, had been acquainted with Mrs. Cumming for a number of years. I had these interviews for the purpose of obtaining, as far as I possibly could, all the facts relating to the past history of Mrs. Cumming's life. — Q. Did you take all the pains you could to ascertain all the antecedents? A. I did. — Q. And I believe you also saw Mr. Ince? A. I also saw Mr. Ince. — Q. What was the substance of the conversation which you had with Mrs. Cumming for the purpose of ascertaining her soundness or unsoundness of mind?

Sir FREDERICK THESIGER.—You read in your opening the whole of Dr. Winslow's report.

Mr. JAMES.—I know I did; but I would rather have this evidence on oath. It will not occupy much time.

*Dr. Winslow.*—Understanding that Mrs. Cumming had an unnatural antipathy to her children, of course I referred to that point. She became a little excited when I mentioned the name of her children, and complained of their unnatural and cruel conduct towards her. She said that previously to Captain Cumming's death, she had been dragged from her house to an asylum, after being put under restraint. — Q. Did she narrate again to you the circumstances of the commission? A. She referred to the circumstances of her being dragged to the York House Asylum, and being put under restraint while she was in the act of getting into a carriage. — Q. Did she then speak to you on the subject of her antipathy to her children? A. Yes, I attempted to ascertain whether I could not reason her out of that antipathy, and I made some excuses for the family. I said it was the husband's act, that it was legally his, and not the children's. She said, "No, it was done by my children. He was my children's tool. He was an old man, and was bed-ridden, and a willing agent in the hands of his daughters." — Q. Do you remember anything being said about some poison which has been charged here as a delusion. Just tell us what she said to you about that? A. She said that some years ago, she had a suspicion that some extraneous substance was in the milk; that her suspicions had been excited by the fact of some fowls having been found dead, and on giving the cat the milk to drink, the cat refused to drink the milk, and that excited her suspicion as to the existence of some poisonous substance in the milk. I endeavoured to reason her out of that impression, but she said that such was the fact, that she had sent the milk to be analyzed, and that Dr. Barnes had assured her that there was poison in it. — Q. What other statement was made? A. I then said, You have alleged that Mrs. Ince had attempted to strangle you, and she said, "Ah! that is one of my delusions." Then, I said, perhaps you may be able to give me a satisfactory explanation of the circumstance referred to, which has led to the impression of your being under a delusion on that point. "One of my delusions?" she said so playfully.

Sir FREDERICK THESIGER.—Will you fix the date of this?

*Dr. Winslow.*—On every interview I had with her I referred to these alleged delusions.

Mr. JAMES.—You alluded to these alleged delusions of which we have heard. It is proper to do so when a medical man is endeavouring to ascertain soundness or unsoundness of mind? A. I did, on every occasion. She said, some time back, pending the proceedings which her children had instituted against her, when her mind was very much irritated and annoyed at Mrs. Ince's proceedings, imagining that they were anxious to confine her again in an asylum, and deprive her of the control over her property, her daughter, Mrs. Ince, came to the house, knocked violently at the door and asked if Mrs. Cumming was in. Without waiting to be announced, she rushed up into her room, threw her arms violently about her neck. Mrs. Cumming made an exclamation, "Oh! dear, you have come to strangle me," or "Have you come to strangle me?" Then, I said, you are not under the impression that your daughter in reality intended to commit any act of the kind? She replied that she was very apprehensive at the time, considering the cruel and unnatural conduct of her children. She did not consider that she was safe, and that no doubt she made an exclamation of the kind. She then observed, that it was a foolish observation for her to make, but in reality she did not believe that her daughter contemplated so brutal an outrage; it was an unguarded, foolish expression of hers, which had been seized hold of and adduced as evidence of her insanity. — Q. In these interviews, am I right in stating that these were the three leading delusions which had been alleged against her: aversion to her children, the question of the poisoning, and the attempt on the part of her daughter to strangle her? A. The medical certificates consigning her to the lunatic asylum referred only to these points. — Q. And also in Dr. Monro's and Sir Alexander Morison's report? A. Yes. — Q. I do not put it invidiously at all, but before ascertaining whether an impression on the mind of a person is a delusion or not, is it not essential to ascertain whether there is the existence of any fact which may be a ground of such delusion? A. It is an important preliminary inquiry. — Q. With reference to the antipathy to her children, are you of opinion that it is a delusion under all the circumstances of the case or not? A. Certainly not.

Sir FREDERICK THESIGER.—But what circumstances?

*Dr. Winslow.*—Under the circumstances she stated.

Mr. JAMES.—Now, I put it thus: Under the circumstances of the commission; of the subsequent proceedings against her, and the conduct of her children, are you of opinion that that really is a delusion or not. A. Certainly not. — Q. Now, state why not, if you please? A. I think that the course which has been pursued towards Mrs. Cumming, has been such as to justify in her mind a natural antipathy and dislike to her children; an antipathy the existence of which may be compatible with a healthy condition of mind. — Q. In one mind there would be an intensity and feeling more than in another, in regard to the conduct of the children? A. It is dependent on the natural constitution of the mind. — Q. With reference to the question of the poisoning, having ascertained the facts, are you of opinion that that is a delusion or not? A. I do not. I think she had good reason for supposing that there might be some extraneous substance in the milk. Being naturally suspicious, and that element in her character having unfortunately been acted upon for a series of years, by the unhappy circumstances of her life, an exaggerated development was given to it; and as she was informed, after her suspicion was roused, that poison was discovered in the milk, after being subjected to chemical analysis, it was a very reasonable and rational suspicion for her to entertain that it was done so designedly, and this led to the existence of the alleged delusion.

The COMMISSIONER.—She had good reason, being naturally suspicious? A. That feature in her character being operated upon unduly by the unhappy circumstances of her life, gave to the fact referred to, an undue and perhaps exaggerated development. Under these circumstances, it was natural for her to imagine—it was within the range of possibility—that poison might have been introduced into her milk.

Mr. JAMES.—A fowl had been killed? A. A fowl, she said, had been discovered dead in the garden, and that of course gave force to her suspicions. She said most distinctly to me on every occasion, on which I adverted to the topic, that she accused no one of an attempt to poison her. — Q. She said so distinctly? A. Distinctly, on every occasion on which I adverted to the topic. — Q. Now, with regard to strangling, the other delusion, which over and over again was made a prominent part in this case, you say she stated her impression was removed? A. She said it was a foolish, unguarded expression of hers. — Q. Did the mode in which she stated that to you, argue sanity or insanity in your mind? A. I think the explanation which I offered in regard to the alleged delusion about the poison, admits of an application to the alleged attempt at strangulation. It is possible she might, being under an apprehension that her daughters were ready at any moment to remove her to an asylum, and being an old lady, not very choice and guarded in the use of her expressions, very likely exclaim, "you have come to strangle me!" and perhaps a few minutes afterwards admit the absurdity of the exclamation. When she explained the fact to me, in the manner just stated, she added, it constituted another illustration of the willingness of her children to seize hold of any unguarded expressions she might use, for the purpose of adducing them as evidence of her insanity. I am now giving you the substance of what she said—of course not in her own language. Having read the examination to which the learned Commissioner subjected Mrs. Cumming, on a former commission, and having heard from Mr. Turner that her mind was deluded in reference to some of her grandchildren, I thought I was in duty bound to examine her upon that point. — Q. Did you direct your attention to that point? A. I did. — Q. It would serve to test her memory, if it did nothing else? A. It tested her memory, as well as her sanity. I told her I understood she was under some very erroneous notions with regard to her grandchildren. — Q. Mrs. Ince's children.

Sir F. Thesiger.—Some of her grandchildren? *Dr. Winslow.*—A. Some of her grandchildren. I said, I had understood she had represented that one was not her grandchild—and that the body of the child had been glazed over after death. She said it was a falsity. I said, " Are you certain that you made no observations that would warrant such a construction?" She replied, it is possible that she might have said it was a handsome-looking corpse, and looked like some of the dolls in the shops in Regent Street; but, beyond that, she was confident she never made any remarks. She might have observed that it was a handsome corpse, and like some of the wax dolls. — Q. You perhaps have seen children—young children, infants, who have died of scarlet fever? A. I have, but not many. — Q. Is there any particular appearance about a child that has died of scarlet fever? A. I think that under these circumstances, the skin has a shiny appearance, from the fact of the disease being a disease of the skin, and the natural exhalation from the surface having been interfered with during the course of the disease. — Q. Does the body of an infant dying from that cause, present a shiny appearance of the skin, being somewhat glazed? A. It does. I have seen it in several instances, and I think it admits of a physiological explanation. — Q. So that the face in the shroud in the coffin would present that shiny, glazed-like appearance. A. To a certain extent it would. —Q. As to her property, did you inquire anything of her? A. I asked her as to her property; she said that it consisted of landed property, household property, situated in Monmouthshire, and her income ranged between 400*l*. and 500*l*. a year. I then asked her some simple questions in arithmetic, apologising for asking questions which might appear very foolish and childish. I asked her the number of shillings in the pound, and other questions, with a view of testing her capacity. I put several questions to her, most of which she replied satisfactorily to; nothing beyond that. I said to her, supposing you were to invest your capital in the purchase of houses, what rate of interest would you expect for such an investment of your property? she replied, that would depend upon the quality of the house. — Q. I believe, without going into it more at length, I may ask this: you took every opportunity within your ability, as a medical man of experience in these matters, to test her as to whether her mind was sound or unsound? A. I did: I never devoted more time or more pains to any case in my life. — Q. She is a person of naturally irritable temperament, is she not? A. So I should imagine. — Q. Is it your opinion that, after all the pains you have taken to ascertain this matter, she is of sound mind? A. Undoubtedly so.

Cross-examined by Sir F. Thesiger.—Labouring under no delusions at all—that is your opinion? A. No delusion that I could discover. — Q. Is it your opinion that a person who labours under any delusion is of sound or unsound mind? A. If a delusion in the proper acceptation of the term is found to exist, undoubtedly it is evidence of unsoundness of mind. — Q. A belief in the existence of a fact which does not exist, is that, in your opinion, proof of delusion? A. Certainly not. — Q. Suppose, for instance, you entertained the notion that I had endeavoured to murder you, would you consider that a delusion or not? A. Not *per se.* — Q. What do you mean by not *per se?* A. Not taken by itself, and without reference to other circumstances which may have induced that belief in my mind. — Q. Supposing that we had met, and that I had offered you some insult, and that afterwards you had entertained the notion that I had endeavoured to murder you, would that, in your opinion, be a delusion? A. No; it may be a false impression, arising from actual circumstances, and not a delusion in the right signification of the word. — Q. Without any other foundation than that an insult had been offered to you—I put it in that way? A. It is possible that it might be only a false impression. — Q. Is it your opinion that "insanity does not admit of being defined—that it is not in the power of any human being to prescribe within the limits of a definition all the peculiar characteristic symptoms of mental derangement?" A. Such is my recorded opinion. — Q. "The malady assumes so many forms, and exhibits itself in such protean shapes, that it is out of our power to give anything the semblance of a correct or safe definition as could be referred to as a standard in doubtful cases of derangement of the mind?" A. Most undoubtedly. — Q. I think the passage I have just read is contained in a book, entitled the *Plea of Insanity in Criminal Cases*, for which I return my thanks, it having been written some years ago and presented to me by Dr. Forbes Winslow. It is dedicated to Sir Frederick Pollock. I mention this fact, because we shall see Dr. Conolly presently. You have spoken about the poison, and you have said she believed that the milk had been poisoned? A. So she said. — Q. Did you apply to Dr. Barnes upon the subject. A. I did. — Q. Your impression, I believe, was, that Dr. Barnes stated that there was acetate of lead in the milk? A. So I believe. — Q. Do you not know that it was Epsom salts, and not acetate of lead? A. So I have subsequently heard. — Q. Would it make any difference, in your opinion, supposing you were now told that she had been informed it was Epsom salts in the milk, and not acetate of lead, or any other poison? A. At the time she told me that she was informed that there was acetate of lead in the milk. — Q. Pardon me, I am putting a different question to you. My question is this—Supposing you had been aware that she had been informed by Dr. Barnes, and repeatedly informed, that there was Epsom salts in the milk, and not poison of any description, would that change your opinion at all? A. Not to any great extent. — Q. But would it change your opinion to any extent? A. I think the fact of her milk having been drugged might, in a woman with a mind constituted like Mrs. Cumming's, and drugged, too, with a substance which in appearance is very much like oxalic acid, convey to her mind a suspicion that there was some foul play going on. It might tend, in her suspicious mind, to create considerable alarm and apprehension. — Q. But you do not understand me, I think. Supposing she was told by her medical man, upon whom she had the greatest reliance, that there was no poison in the milk at all, but that it was merely Epsom salts, and supposing she afterwards entertained the notion that there had been an attempt to poison her, should you consider that that was a delusion or not? A. No, I should not think it was a delusion in the proper acceptation of the term. — Q. Why not in the proper acceptation of the term? What do you call the proper acceptation of the term? A. I do not think that it was the creation of the mind *de novo.* — Q. A creation *de novo!* What do you mean? — A. It was an idea which followed actual circumstances. It might have been a mere mistake of hers. Having been told that the milk was drugged, she might have had an apprehension that some attempt was made upon her life, and so believe the fact. This is possible, without its justifying the belief, that she was therefore of unsound mind. — Q I think we are at cross purposes. I am taking for granted that whatever impression was originally made in her mind, that impression was removed by the communication to her, that there was nothing but Epsom salts, which are harmless to a certain extent, and that she, notwithstanding she was told to the contrary by a person on whom she could rely, she still persisted in believing that there was poison in the milk? A. Taking the case in all its circumstances, viewing the natural temperament of Mrs. Cumming, and the peculiar constitution of her mind, and all the prior unhappy circumstances of her life, I should not be at all surprised if she were to misstate and exaggerate actual circumstances, without the notion being the result of a disordered condition of mind.—Q. You think so. A. I do.—Q. Do you mean to say, that if a person has once entertained a suspicion of a particular fact, and that suspicion is entirely removed from her mind, and the suspicion afterwards recurs, that there is a justification for the suspicion, although her mind had been completely cleared of any such impression for a considerable time? A. I think you cannot form a correct opinion of the matter without reference to all the circumstances of the case. — Q. Then that is your opinion? A. That is my opinion. — Q. Then if at one time she entertained a suspicion, no matter how it may have been removed, the recurrence of that does not prove a delusion? A. If she once entertained the suspicion that poison was infused into her milk, and she was informed that, instead of poison, the milk had been drugged, no matter how innocent the drug might in reality be, it might, in a mind constituted like Mrs. Cumming's, viewing all the prior events of her life, and her constitution of mind, give rise to an impression of poison, which impression could be co-existent with a healthy mind.— Q. With a healthy mind? A. Yes. — Q. Then you consider that a person who has entertained suspicions, which suspicions had been entirely removed, and those suspicions occurring again, may be in a sound and healthy mind? A. A person may be under an erroneous impression, and may arrive at a false result at one period, and to a certain extent it may be justified; that erroneous impression may subsequently occur to the mind from actual circumstances. — Q. May subsequently occur to the mind from actual circumstances? A. Yes. — Q. That is your opinion? A. It is. — Q. I am putting where the impression is entirely removed? A. It is possible even under these circumstances. — Q. And no circumstance recurring to remove the false impression. Yet that impression recurring, and recurring with great strength, so as to induce the belief that a great crime has been committed, do you consider that that is explicable upon the ground of the previous circumstances and character of the party? A. I think you cannot arrive at a right result without viewing the case of Mrs. Cumming in all its circumstances.—Q. I am not asking you as to the case of Mrs.Cumming? A. Supposing an impression to have once existed, and for the party to have been undeceived, I can imagine that there would be a recurrence under peculiar circumstances of the false impression, such recurrence being consistent with the existence of a healthy state of mind; the impression need not necessarily be a delusive one. — Q. Then I suppose when once an erroneous impression is entertained, that erroneous impression depending on slight circumstances we will say, but still an erroneous impression, when once that erroneous impression is entertained, its constant recurrence does not indicate any unsoundness of mind at all? A. Erroneous impressions are often entertained by very healthy vigorous minds. — Q. But an erroneous impression from slight circumstances, which impression is entirely removed? A. I will illustrate what I mean. Take the case of A, B, and C, as I am not permitted to mention Mrs. Cumming's case—take the case of a person under a belief that certain poisonous ingredients had been infused into an article of diet, prior circumstances naturally exciting suspicion in the party's mind, and an apprehension that something of the kind might occur, it is possible under those circumstances for the person to believe when so informed that he was under a false impression as to the fact with reference to the poison. Subsequent circumstances might, however, occur with regard to the same individual, which would revive the previous false impression. The party might argue, and argue reasonably, "I was under such an impression at a certain period. I was subsequently told it was a false impression. Circumstances have since occurred which certainly do convince my mind that it is within the range of possibility that the thing might have actually occurred. Under these circumstances, the party might believe in the existence of the fact, that belief being consistent with the presence of soundness of mind. — Q. You have put the case where the impression continued, and where there are fresh circumstances. I put the case where there are no fresh circumstances to revive the impression, that impression reviving to the extent of supposing that persons were prepared to poison her. A. No, I do not think, even in the hypothetical case which you have suggested, that under those circumstances we should be justified in believing the impression to be necessarily a disordered creation of the mind. — Q. Now, I will put another case to you. Supposing a person had employed an attorney, and has confided her interests entirely to him—that that attorney had been in the habit of receiving money, and of attending from time to time, and performing the part of a faithful agent, and that the principal was aware of the faithful performance of duty by the agent. Supposing, without any new circumstances occurring, she was at a subsequent time to entertain the impression that the agent had robbed her of her money ; that he had robbed her of everything she possessed, and left her penniless, and that he had never rendered her any account at all, should you be of opinion that that indicated a sound or an unsound state of mind? A. Those notions, I think, are consistent with soundness of mind, particularly if occurring to a person naturally of suspicious temperament. — Q. In a suspicious mind? A. Yes. — Q. But observe, this suspicious mind has been confiding for a considerable time, and relying confidently upon the party, and then suddenly, and without any reason at all, turns round, and charges that party with having robbed and plundered her, with having rendered no account, and having enriched himself by the spoils of her fortune? A. That shows great caprice, but it is possible that such a feeling might exist apart from insanity.— Q. Is that a "creation *de novo*," in your judgment? A. Certainly not. — Q. That is not a "creation *de novo?*" A. No; it is an idea arising out of actual circumstances. — Q. I put my case guardedly. I put this case. The utmost confidence is expressed from day to day, and manifested in every possible way; then there is suddenly a new idea started up that the party so trusted had been false in every respect, had plundered and left the person penniless, and was revelling in the spoils of that fortune. A. That would show great caprice and an ill-regulated mind, and a mind suspicious without reasonable foundation. — Q. Then you will tell me what you mean by a "creation *de novo*." A. An impression or notion arising in the mind apart from the actual circumstances of life. — Q. That is from the existing circumstances of life? A. From the existing circumstances of life. — Q. That is your "creation *de novo?*" A. Yes, and it is, to a certain extent, a scientific test of delusive impressions. — Q. Now I will put this case. Suppose he had faithfully rendered an account, and money had been paid over, and a belief had been entertained that no accounts at all had been rendered and that no money at all had been paid? A. The impression may have resulted from a mere failure of memory. — Q. Do you suppose that such a person would be competent to the management of his affairs? A. That incompetency, if the incompetency existed, might be the result of advanced age, or from careless habits of business, or from a natural indisposition to attend to the ordinary business matters of life. It need not necessarily be an incapacity arising from unsoundness of mind. — Q. But I put the case of a person. A. I think it is possible that such a condition might exist apart from actual insanity or unsoundness of mind. — Q. Apart from a capacity to manage the affairs of life? A. No; I draw a distinction between the incapacity and natural decay, which is, in many cases, the inevitable result of old age, and the incapacity which is clearly the offspring of insanity. — Q. But I am putting it to you now—do not vary the ground. I put a distinct question, whether such a person as I have described would, in your discreet judgment, be competent to the management of his affairs? A. In the proper acceptation of the term, such an amount of incapacity would not necessarily indicate unsoundness of mind or legal incompetency to manage the business affairs of life. — Q. Would such a person be competent to the management of his affairs? A. *Legally* speaking, certainly. — Q. *Actually* speaking would he? A. There are many sane persons who are incapable of managing their affairs and who leave all their business matters to their solicitors. — Q. You are running away from my question. I have put a particular case to you, which is clear and distinct. I have put the case of a person who had received from his attorneys accounts of monies faithful and true throughout, and then believing that no accounts had been rendered and that no money had been paid. A. I should say that such was a strange, but not necessarily an unsound condition of mind. — Q. Would you consider such a person—a person who had received money and accounts, and yet believed that he had not received the money and had not received the accounts, a person capable of managing his own affairs? A. I should say that such was a strange and unnatural condition of mind, but not necessarily an unsound condition. — Q. I understand in one sense what a natural mind is. A. You may have such an amount of incapacity coexisting and consistent with sanity. That is what I mean. — Q. But I put a particular case to you. Dr. Winslow, I know you are a match for me, but do confine yourself to the case I put. It is a very distinct one—it is this. I ask you, in your judgment (of course appreciating your character), whether such a person is, in your judgment, competent to the management of his affairs? A. I think the world would say not. — Q. What would *you* say—do you agree with the world or not? A. I have seen cases of incapacity to the extent referred to by yourself, associated with perfect soundness of mind. — Q. You have told us that the world would have an opinion upon that subject. Do you agree with the world or do you differ from the world? A. I do not bow to the opinion of the world as an authority upon points of abstract science. — Q. Then you look down upon the opinion of the world? A. Certainly not. I consider the question as one of science. When I am asked whether, in a given case, there is an incapacity to manage property, my object is to ascertain whether that incapacity is the necessary result of a *diseased mind* or whether it is a *natural incapacity arising from old age, decay of nature,* or *from ignorance of the ways of the world and a natural careless indifference to the affairs of life.* That, in my opinion, is the scientific distinction. — Q. I am putting a case in which there can be no doubt about the moneys being paid and the accounts rendered, and in which the party believes that no money has been paid and that no accounts have been rendered, would you require to ascertain all the circumstances respecting a person's mind before you could come to a conclusion whether such a person was capable of managing his affairs or not? A. I think we could not take as a rule an isolated feature in a particular case and draw safe conclusions from it. I should be very loth to say, if a case of great incapacity was established, that that incapacity was necessarily the incapacity of an unsound mind. Many men of mature age and vigour of mind are not of business habits, and are not capable of managing their property. — Q. There are circumstances which do not depend upon business habits at all. I put a plain and palpable case to you. A. I think in the case put by you the circumstances of course would be suspicious, but such an amount of incapacity might coexist with soundness of mind. — Q. Then you are of opinion that the case I have put to you is perfectly consistent with entire soundness of mind? A. It may be so; I have no doubt upon the point. — Q. Now I will take the case of strangling. Suppose there is no truth whatever in the daughter throwing her arms round her mother's neck, or her mother having been at all alarmed by her appearance in the Edgeware-road, should you consider that the absence of those facts would be sufficient to lead you to the conclusion that she was labouring under a delusion when she stated her daughter was going to strangle her? A. If the facts as stated by her were not facts, certainly. — Q. Well, then, you would consider her under a delusion? A. It is possible (admitting the truth of what you say), that her mind might be unsound upon that point. — Q. Did she tell you, at any of these interviews with her, that Mr. Haynes had poisoned her fowls? A. She said she understood that some of her fowls had been poisoned by the order of Mr. Haynes. — Q. It was in allusion to this poisoning of the fowls and of the milk it was in? A. Yes. With reference to the alleged delusions with regard to the poison, perhaps I may be allowed to state that one of my reasons for not considering it to be such at the time was this, that if she had laboured under a delusion that an attempt had been made to poison her, if it had been in reality a delusion—the product of a disordered mind,—and the delusion in question had been persistent up to this period, still having its influence upon her mind, according to my experience, admitting it to have been an insane delusion, it would not have confined itself to an epoch or to an individual, but so terrible would be its influence upon the mind, even at this moment——

Sir F. Thesiger.—Stop, Dr. Winslow, you are giving us a very long speech.

Mr. James.—He is answering your question, and I beg that he may be allowed to finish his answer.

Sir F. Thesiger.—This is no answer to my question—this is something which he volunteers. — Q. I cannot permit you to go on in the way you were doing. I will ask you this question—suppose, for instance, with regard to the strangling, she stated sometimes that it was at one place, and sometimes at another place; should you consider that that was an indication of a delusion? A. No, not of itself, it might arise from a failure of memory. — Q. Although there were no facts to warrant it at all? A. Of course, if there were no facts to warrant the impression, that would be a circumstance justifying the suspicion of unsoundness of mind. Q. Suppose the only fact was the visit of her daughter to her, and she received her daughter with great kindness, should you consider that fact sufficient to warrant an impression that her daughter had attempted to strangle her? A. Certainly not. — Q. Then I understand it to be your opinion that if a person entertained any belief of a fact which is entirely unfounded, that that is a delusion, and that if the party acts upon that impression, or belief, that indicates unsoundness of mind. A. I did not follow you. — Q. If a person believes in the existence of any particular fact, without any foundation for it, and acts upon the belief of that fact, does, or does it not, indicate unsoundness of mind? A. Not necessarily so.

Re-examined by Mr. James.—You have been asked whether it is not difficult to define insanity: I believe you adhere to the opinion which you have always given —that it is a most difficult thing to define? A. It is undefinable. — Q. It may exist in a thousand forms, may it not? A. Yes; in a thousand forms. — Q. As in the diseases of the body? A. As in the diseases of the body. — Q. Is it a test of insanity to suspect an attorney of cheating you? A. I should be sorry to propagate such an opinion. — Q. Suppose an attorney asserts to a person that he has only received a certain amount of rent, and that he has rendered accounts, and faithful accounts, would it be a test of insanity for a person to suspect that that was not true, although the attorney made that assertion? A. Certainly not. — Q. Are suspicions necessarily delusions? A. They are not. — Q. Is a jealous person, for instance, a person of unsound mind—a person who suspects his wife without cause? A. Such a suspicion may co-exist with perfect soundness of mind. — Q. Extreme jealousy of a most virtuous wife may co-exist with perfect soundness of mind? A. It does sometimes. — Q. And suspicion? A. And suspicion. — Q. Now, with reference to these three delusions, I will take first, the strangling; has she not stated that fact to you, of the strangling, without stating also the concomitant circumstances at the interview from which she derived the impression? A. Never.

Sir F. Thesiger.—Will you have the goodness to ask Dr. Winslow the last time he saw her.

Mr. James.—When was the last time you saw her? — A. On the 6th of this month; the day before the inquisition. — Q. Has she ever stated to you the fact of this strangling, without stating to you the concomitant circumstances which induced the belief in her mind? A. She never did. — Q. Did she ever state to you the fact of the poison without also stating the concomitant circumstances attending it? A. Never. — Q. You have been asked by my learned friend, Sir F. Thesiger, as to the discovery of Epsom salts in the milk, and of acetate of lead being found. That occurred at the same time that a fowl was found dead.

Sir F. Thesiger.—There is not any proof of that whatever.

Mr. James.—You will allow me to put it hypothetically. Q. My learned friend put to you the case of the Epsom salts in the milk, only assuming it to be the fact that a fowl was poisoned, and that deadly poison was found in a paper in a fowl-house; that occurring at the same time that the milk was found to be drugged, though with a harmless drug, does that justify her forming the opinion which she expressed? A. I think it would. — Q. Must not the impression on her mind be taken with all the circumstances attending it? A. With all the circumstances attending it. — Q. And you think that the fowl being found dead, and poison being found in a paper, and contemporaneous drugging of the milk, even by a harmless substance, ought to be taken into consideration in considering the question whether or no she was under a delusion? A. Most certainly. — Q. And I think you say that, upon every occasion when she mentioned the circumstance, she always referred to the facts attending it? A. Yes, she always stated the concomitant circumstances. — Q. Upon giving an answer which was stopped by Sir Frederick Thesiger, but which appeared to me to be a most able and philosophical one, will you have the goodness to repeat now? A. What I wished to explain was this—that if this belief in the poison had been a symptom of a diseased mind, admitting it to be a delusion, and persistent, being still in existence, so terrible is the influence which delusions of this kind exercise over the conduct and actions of insane persons, that within the records of my experience, I have seen no case whatever where it would be confined to one epoch, or to any one or two individuals, but it would influence generally the conduct and character of the patients, and they would continue in the belief that their servants were trying to poison them, and would be suspicious, and perhaps refuse food altogether. I never knew any instance in which that was not the case. If Mrs. Cumming, at this moment, laboured under a delusion of the kind, in all probability, and I may say almost to a certainty, she would decline her food in consequence of her being suspicious that Mrs. Moore, or some of the servants about her, might poison her. — Q. She relates it to you as a fact that had occurred, and not as a present delusion haunting her mind? A. No; she relates it as a fact that had occurred. — Q. Does it not make a great difference in the question whether a person narrates it as a fact that had occurred and as an impression that had existed, although a wrong one—is there not a great distinction between that and a present delusion haunting the mind? A. A most material distinction. If the delusion existed at the time when the poison was alleged to be introduced into the milk, it is quite clear that it does not exist at this moment. — Q. Is it not, therefore, rather the character of an impression, to some extent false, from the facts from which once existed in the mind, than a present delusion haunting the mind? A. Yes. — Q. When she spoke to you of her aversion and antipathy to her children, did she not narrate to you at each time that which had been—whether properly or not—assigned as the cause of that aversion? A. She did. She referred to her having been seized and carried before a magistrate on the charge of perjury; to her having been followed and dodged about by policemen, and to a variety of circumstances in reference to the alleged unnatural conduct of her children, she always coupled these circumstances with the explanation of the impressions which are alleged to be delusions. — Q. To go back from this psychological discussion, is it your opinion, from the best judgment you can form, that she is of sound mind? A. I have no hesitation in saying that in all my experience of doubtful cases of insanity, I never met with, or examined a patient where the result to my mind has been more clear and satisfactory.

*Samuel Ashwell, Esq., M.D.*, sworn, examined by Mr. James.—You are, as we know, a physician, and I believe you are practising in Grafton-street? A. Yes.— Q. You were for many years a lecturer at Guy's Hospital? A. I was. — Q. I believe you have not directed your attention exclusively to cases of insanity? A. No. — Q. Has it occurred to you in the course of your practice to see many cases of insanity? A. Many throughout the course of my practice. — Q. And have you seen in the course of your practice very many cases of female insanity? A. Certainly. — Q. I believe you were requested to see this lady? A. Yes, I was. — Q. When did you see her? You saw her yesterday, I believe? A. Yesterday. — Q. How long were you with her? A. I think nearly an hour. — Q. Did you see her alone? Did you desire to be alone with her? A. I was introduced by Dr. Hale, who immediately left the room, and with the exception of a servant coming in two or three times, I saw her alone. — Q. There was nothing to interfere with her examination? A. Nothing at all. — Q. And you were with her an hour? A. Nearly an hour. — Q. You have heard, I presume, that the chief delusions which have been alleged in this inquiry against her, were an aversion to her children, the fact of her believing there had been an attempt to poison her, and the attempt, or alleged attempt, which she said Mrs. Ince had made to strangle her? A. Yes. — Q. I believe you have read a good deal of the evidence. The case had interested you, I suppose? A. Yes, I had; the case had interested me, and I had read a great deal. — Q. Is the opinion which you are about to give founded entirely upon the examination and your personal interview with her? A. Absolutely. — Q. Did you converse with her? A. Very fully. — Q. As a medical man did you converse on these subjects? A. Very fully. — Q. What topic did you take up first of the alleged delusions? A. I began the conversation by asking her how it was that the delusion or mistake arose about her children; she immediately said there was neither delusion nor mistake, that her feelings in reference to them were the result of their conduct towards her, that there was no delusion in her mind about it. — Q. She said so? A. She said so. She then went through what I suppose I need not detail here, the whole of their conduct towards her. She narrated the whole of their conduct towards her in reference to the commission. — Q. The former commission? A. The former commission—the transaction about the perjury, on which she dwelt most vehemently. — Q. That is her arrest on the charge of perjury? A. Yes, the arrest, and her determination to act as she thought right in the distribution of her property. I alluded to the unfavourable impression it would make if she diverted that property from her natural heirs. She immediately said, "It would be so if I had not good ground for it." Those are her very words. She then said there were other people who did the same, who did not leave their property in the ordinary way, but whose intellect was not therefore called in question. — Q. She said so? A. She said so. I then said, "Might you not leave some to the grandchildren?" — Q. To the grandchildren? A. To the grandchildren—to which she replied, "that she did not feel inclined to do so." — Q. Did you speak to her about the case of poisoning? Did you take that next in order, or which did you take in order? A. I then commenced about the case of poisoning, and I said, I had heard that she had been labouring under delusion as to an attempt having been made to poison her. She said, "There was a mistake about that." Those were the words she used. She said there was a mistake about that. She thought she had sufficient ground at all events at the time. — Q. Did she tell you about the analysis? A. She told me that the analysis had discovered that there was acetate of lead in the food. She said it had been discovered by Dr. Barnes that there was acetate of lead, or some other poison, from eating of which the fowl had died, and that there was sulphate of magnesia in the milk presented to herself. — Q. That is Epsom salts, is it not? A. Epsom salts.

The Commissioner.—In the milk presented? A. To herself. She very minutely recapitulated all the circumstances as to the situation of the fowl-yard, the poultry-yard, and the conversation which had taken place between herself and servant.

Mr. James.—Did you then advert to the strangling at all? A. I said, "I should like you to tell me about the strangling." She told me that she had been alarmed at the suddenness with which the movement towards her had been made by her daughter, and for the moment she said, "I might have supposed I was going to be strangled, so sudden and so violent was it." I then said, "Of course you do not entertain that opinion now."

The Commissioner.—She said the movement towards her was so sudden. A. So sudden, and I think she added, unexpected. I said, "Of course you do not entertain that notion now." "Certainly not; it was clearly a mistake on my part."

Mr. James.—Did you then question her about her property? A. She then told me about her property. I said I should like to hear something about her property. "Will you tell me its amount, its yearly value." She said, "It is worth 500*l.* or 600*l.* a year."

A Juryman.—Now? A. Yes, now, as far as I understood her. "Of course," she said, "it would have been worth much more, but for the expense of these dreadful proceedings." She used the word "dreadful," or something to that effect. — Q. From the statement which she made to you, whether her aversion to her children may be rightly founded or not, should you call that a delusion? A. Certainly not; she made the distinction herself, it cannot be a delusion; it may be wicked, but with that the law has nothing to do. — Q. She said so? A. She said so. — Q. Is an aversion entertained by a person towards her children, who, from a course of conduct they may think have acted most unkindly or most ungrateful to them, is that a delusion? A. Certainly not. — Q. It is a sentiment of the mind? A. Certainly. — Q. Not a delusion? A. Not a delusion. — Q. Does the intensity of the aversion show any evidence of a delusion? A. I think that depends upon the character of the mind. A person of quiet temperament would in all probability be satisfied with a moderate expression of aversion, a person of strong feeling would take a more emphatic form of expression. — Q. With reference to this person did you find that there is any delusion as to the poisoning at present dwelling upon her mind? A. Not at all. — Q. Am I right in stating it is an impression of something past? A. I thought it was an historical expression past and gone. — Q. Not haunting her mind at the present time? A. No. — Q. Have you any doubt at all about it? A. The only doubt I have arises from this fact, that she seemed to hope that she should not be dragged away again. — Q. Did she express to you any dread of a lunatic asylum? A. Yes, she said "I hope the commission will terminate in my favour; if it does not, I shall probably not live three or four months."— Q. She said so? A. She said so. She said, "The shock will be too much for me." — Q. Do you agree with Dr. Winslow, that a delusion is the test of insanity or the creation of a diseased mind, that one of the symptoms of that delusion is its constantly haunting the mind and influencing their actions? A. Certainly. — Q. Did you find any delusion exist? A. Not at all. If I had seen her without any reference to the commission, I should have seen her, as I should have seen any other lady patient, and left her with a full impression that she was perfectly sane in every particular. — Q. Is it your opinion that she is of sound mind? A. Oh, certainly. I do not mean to say that she is a woman of the very strongest mind, but of perfectly sound mind, and I think it very likely, if she were to have questions brusquely put to her, and thirteen or fourteen people present, she might show some alarm. — Q. She is very old and feeble? A. She told me she was seventy years only. — Q. There is a distinction between a memory somewhat impaired and a diseased mind? A. Oh, yes. — Q. Have you not met any people of her age and position, who cannot give you an account of their property when put before them, who yet would be perfectly sane enough to dispose of it? A. Many; but she has a great deal more than that. — Q. I am putting a partial failure of memory in a person of her age who has suffered so much, as no test of insanity? A. Certainly not. — Q. Then it is your opinion that she is of sound mind? A. She described to me very accurately the circumstances of her being dragged away from her house in 1846; she described the two nurses, whom she called two great, large women, sitting by her side. — Q. That was effort of memory? A. That was an effort of memory; and the manner in which she was treated at Dr. Millengen's: she said she never was treated as an insane person while she was there. — Q. Is it your opinion she is of sound mind? A. Certainly.

Examined by the Commissioner.—Q. Did she seem to be under the impression of being taken to an asylum? Did you attempt to soothe her? A. I told her I did not think there was much probability of her being taken to an asylum. I went as far as this, I said: "Even if the commission should be unfavourable, there is not absolute necessity;" but she immediately said—"That will be equally fatal, giving my property, or withholding my property as I think right."

Examined by the Jury.—With respect to the strangling, you say that idea has gone from her mind entirely? A. I thought so. — Q. Did you take any opportunity of expressing to her that it might have been over-affection? A. I did not put it so. — Q. Did you say, As it is a mistake, perhaps you will have a more kind feeling towards her daughter? A. No.

Mr. James.—The great object was to hear her state her views? A. Exactly.

A Juryman.—This lady is very much affected with paralysis, is she not? A. I should say she is very much out of health indeed. — Q. Is there any fear of her being seized suddenly with apoplexy? A. I think there is. — Q. Do you think the way she has been treated now, being in the habit of taking wine and brandy, and other stimulants—do you not think, by being under that treatment, it is very likely to bring on apoplexy very suddenly, and cause sudden death? A. My visit would not justify me in forming any opinion. She was not under the influence of anything at the time when I saw her yesterday; but I should think, from the flabby condition of her flesh, without the moderate use of stimulants, she would sink and die. — Q. You think of the two evils, they choose the least? A. I do not think I am called on to speak to that alternative. — Q. Did she know you were coming? A. I think Dr. Hale told her, but it could hardly have been a minute or two before. — Q. In your opinion, may a person under strong delusions, and having made unfounded statements—is such a person, knowing she would be under the examination of a jury, capable of being tutored to conceal her delusion, and qualify any statements she may have made? A. I think she was totally incapable of that kind of delusion; but if so, it was an insane delusion.

The Commissioner.—After a certain time? A. I should say, after it has established itself in the mind. After an insane delusion has established itself in the mind, I think you could not tutor such a patient to receive safely the visits of anybody. — Q. You could tutor her for a time, a very short time? A. I doubt it. — Q. It would not be a permanent delusion? A. No. — Q. Did you ever see a patient with delusions, when you did not discover them for some time. A. I have never seen a patient with an insane delusion, where, if you approached or alluded to that delusion, tutor him as you may, you could get away from him the effect of that delusion.

A Juryman.—Do you think if she thought it was affection on the part of her daughter, when she hung about her neck, it would alter her mind? A. Not now—I think her feeling is too deeply rooted. — Q. If she thought it was affection, it would not alter her? A. Well, my opinion is not worth much on that point. In conversation with Mrs. Cumming, her mind seemed so thoroughly made up, that nothing I think could alter it very much.

Another JURYMAN.—But her mind could not have been thoroughly made up when she stated she was mistaken. — A. I do not think, in reference to that absolute, single, isolated transaction of the strangling, Mrs. Cumming's mind rests upon that only—she related to me a long series of circumstances. — Q. But on that point alone she stated to you, though she apprehended she was about to be strangled at that moment, she had since found she was mistaken about it. — A. I think that impression is removed. — Q. And you think that there is no cause of enmity now? A. Not at all; I do not think it has anything to do with the enmity.

The COMMISSIONER.—It rests on the other conduct which she mentioned? A. Decidedly; and she made a very marked distinction herself. — Q. You did not endeavour to convince her the issuing of a Commission of Lunacy was not a bad act on the part of any one? A. I think if I had, I should have had some difficulty to convince her; she told me it had cost three or four thousand pounds. — Q. Did you endeavour to convince her it was for her own benefit. You do not consider a Commission of Lunacy a diabolical act? A. Not an unmitigated evil. — Q. Then I suppose you would say it is for good? A. Yes. — Q. Did you endeavour to convince her mind there was no evil—that it was an attempt to find out whether she was of right mind or not—not for her injury, but for her benefit? A. I think I should have been convinced of that myself, which I was not. — Q. It is not an evil of the daughters commencing the Commission of Lunacy, unless it is proved to be one? A. Exactly. — Q. Not having been proved to be one, yet did you attempt to satisfy her mind upon it? A. Not at all.—Q. Did you find out how recently she had found out she was mistaken about the strangling? A. No, I did not; it seemed to have passed from her mind entirely, she only referred to it as a past circumstance. — Q. You were not aware she had very recently adverted to it? A. Yes; I alluded to these three great delusions I had heard, and she treated them all as past things, and smiled about them, and said that upon this the commission was founded of course; she said a great deal more than I can detail now.

*John Conolly, Esq., M.D.*, examined by Mr. JAMES.—You have devoted much of your attention to the study of mental disease? A. I have.—Q. You are physician of the asylum at Hanwell, and have also establishments under your care? A. Yes. — Q. When did you first see this lady? A. I first saw her in September 1846. Q. At the time of the last Commission. — A. At the time of the last Commission I saw her at York House. — Q. Did you examine her then? A. I had an interview of two hours with her, on the 6th of December, and a second interview on the 18th. — Q. I believe you were prepared on that occasion, had the case gone on, to have given your evidence on her behalf? A. I was prepared to do so. — Q. From the examination you made of her you were then prepared to have given your evidence on her behalf; I presume I am justified in stating you were of opinion she was not of unsound mind at that time? A. I was of opinion that she was not of unsound mind. — Q. When did you see her again? A. I saw her when the Commission was resumed, after its adjournment at the Horns Tavern, on the 21st and 22nd of September. — Q. From all you saw of her at that time, the examination you made of her, the opportunity you had of seeing her, were you prepared to have given your evidence then that she was not of unsound mind? A. Quite so. — Q. When did you see her again, with reference to these proceedings? On Monday, December 1st, 1851. — Q. You have heard, I presume, that she has been seen by a great many medical men, and submitted to a great many examinations, in the course of the investigation; now, if these alleged delusions existed in her mind, could she be tutored so as in any way to conceal them? A. I think it quite impossible that she could; many patients will conceal their delusions, that is, they will not spontaneously avow them; they will learn to do that; but if you examine and touch on the delusion, with a little care and patience, one never fails to bring it out. — Q. And you have heard the gentlemen who have gone and submitted her to examination on both sides, have at once put to her the alleged delusions, and brought the mind to the topic distinctly? A. I believe, repeatedly, it has been done. — Q. Assuming the existence of a delusion as the result of a diseased mind, and the mind brought to the topic, if it is the result of disease, it is not manifest at once? A. Perfectly so. — Q. So that if you are unaware of the delusion of the patient and speak in an hospital, or elsewhere, and do not touch on the particular diseased chord, you may leave that person under the impression that they are not insane? A. Undoubtedly. — Q. But if you do touch upon the delusion you have discovered the disease; is not that so? A. Almost invariably, I should say. — Q. Then is it your decided opinion that this lady —I use the expression which has been used or insinuated—could not have been tutored so as to suppress any evidence of insanity in these conversations? A. I do not think it possible. — Q. You saw her, I think you were saying, upon the 1st of December? A. Upon the 1st of December. — Q. I will not take you at any length through the dates; how often have you seen her altogether? A. Eight times, including her visits here, and the visits when I attended the Jury to her house. — Q. Were you with the Jury on both occasions? A. On both occasions. — Q. And you heard all the statements which she made to the Jury? A. Yes. — Q. You have, yourself, examined her mind with a view of testing it? A. I have done so. — Q. Frequently? A. Not frequently. — Q. How often? A. On the subject of the delusions, not frequently, because I was not aware of them until recently; when I first saw her some of those delusions did not exist, circumstances had not occurred. — Q. When you saw her in 1846, the circumstance of the poison and Dr. Barnes had not occurred? A. No. — Q. And I believe the impression she is said to have expressed herself, as to her daughter having attempted to strangle her, that had not occurred in 1846? A. That had not occurred. — Q. Have you examined her upon these which are alleged to be delusions, the aversion to her children, the alleged attempt to poison her, and the strangling? A. I have examined her on all these points. — Q. With reference to the aversion for her children, what does she state to you generally upon that point? A. Her statement now is quite consistent with her statement in 1846. She considers she was treated with great harshness, great want of feeling; that she was taken unnecessarily, and violently, to a lunatic asylum. In 1846 it had occurred once, and now it has occurred twice; and this in addition to various little cases of neglect, which she sometimes alluded to. These are assigned as reasons for believing that her children have no affection for her, and the things which seem to have alienated her affections from them. — Q. Am I right, do you agree with other gentlemen who have given their opinion, that an aversion to children entertained more strongly by one mind than another, if there exists some foundation for it, is it a delusion, or an intensity of feeling? A. Merely an intensity of feeling. — Q. Is it a delusion at all as the test or as the creation of a diseased mind. I mean the strength of the aversion? A. Not where it is founded on any real cause. — Q. With reference to the poisoning, when you have put to her that which is alleged to be her delusion, has she stated to you the facts always from which she derived that impression? A. She has alluded to them repeatedly; her impression being that the object has been for a long time to get possession of her money; and now they would be glad if her life were sacrificed that the same end might be accomplished. These expressions are frequently used by her. — Q. Now, the strangling? A. With respect to the strangling, she has given a very simple account of it; that when her daughter had not seen her for some time, when they were not on good terms, her daughter suddenly rushed up-stairs, threw her arms about her neck, with a violent expression of feeling, and that it agitated and alarmed her, and that she thought her daughter intended to injure or strangle her. But the idea appears to have entirely gone away from her mind as a mere erroneous impression at the time. — Q. Do you find either of those delusions existing or haunting the mind now? A. Not at all. — Q. And is not that one of the tests of delusion of an insane mind, that it haunts the mind and influences their actions? A. Certainly. — Q. And you find neither of them? A. I believe neither of them exist. — Q. The aversion to her children is not an aversion, it is an intensity of feeling? A. An intensity of feeling; and the circumstances which are known to have taken place are in my mind sufficient to account for it. — Q. Do you agree with Dr. Ashwell in the opinion he gave, that they are rather historical impressions than present delusions haunting the mind? A. Entirely. — Q. I believe you have taken great pains with this case, and given great attention to it? A. I have taken much pains both in 1846 and on recent occasions. — Q. Is it your opinion that she is of sound or unsound mind? A. I consider her of sound mind. — Q. Do you give a decided opinion? A. A decided opinion. I never discovered in her any incoherence, or any symptom of a disordered understanding. — Q. Perhaps you will allow me to ask these questions:—You are a physician of the Hanwell Asylum; if she were in the Hanwell Asylum, would you retain her in that asylum as a patient of unsound mind? A. Certainly not; I should recommend her for discharge. — Q. That is a public institution of which you are the public officer? A. Yes.

Cross-examined by Sir. F. THESIGER.—You have a private asylum of your own? A. I have. — Q. What is that? A. Where I live, on a very small scale; I take a few patients at my own house. — Q. How many? A. Five or six; never more. — Q. That is, of course, under the control of the Commissioners of Lunacy. A. Entirely. I ought to state, that I am partly proprietor of another, where there are about twenty received. — Q. Where is that? A. At the village of Hayward End. — Q. Is it your opinion "that all well conducted asylums have now become places of protection, abounding in the means of diverting the thoughts and calming morbid excitement, and soothing the depressed, and rousing the apathetic, and restraining the lower propensities of the insane, and restoring the control of reason?" A. All *well conducted asylums*. — Q. There are asylums under the control of the Commissioners of Lunacy? A. Yes; nearly all; there are one or two exceptions—Bedlam is one. — Q. Is it your opinion, that if there is something in the character of a party's mind, it is not only the dangerous lunatic who requires to be placed in an asylum, but the rule for safe general guidance must have a wider extent? A. Certainly. — Q. Is it your opinion "that if there is something in the character of a party's mind which renders him unable to take care of himself and his property, or which is incompatible with his personal safety, or that of others, or with the security of his property, or that of others, or with his own comfort or well doing, if left to himself unprotected, that he ought to be carefully watched after in a lunatic asylum?" A. I think so.

Mr. JAMES.—Do you put the pamphlet in?

Sir F. THESIGER.—No.

Mr. JAMES.—I should like to see it.

Sir F. THESIGER.—I am reading now from a pamphlet which you did me the honour to send me, and for which I return you my thanks, in which, with reference to the Agapemone case, you found fault with the opinion of the Lord Chief Baron, as to the ground of there being no right to confine lunatics, except those dangerous to themselves or to others. — Q. I think you state that nothing is more clear or certain, that that interference is not only justifiable but absolutely necessary in a great many cases, in which neither the person of the lunatic nor that of others is in any way endangered by his malady? A. Yes; undoubtedly. — Q. "Is it your opinion that there are many forms of unsound mind, which, although for a long time unattended with actual danger to the lunatic or others, lead to consequences so intolerable, that an asylum must be resorted to relief?" A. Yes. — Q. And amongst others, are there delusions as to property, as to money owing or withheld; is that your opinion? A. Yes.

A JURYMAN.—If it is evidence, will you have the goodness to mark the passages?

Sir. F. THESIGER.—I have marked them.

A JURYMAN.—They are the settled opinion of Dr. Conolly, on oath I take it?

Sir F. THESIGER.—Just so. I do not put it in.

The Witness.—Of course I answer the question in the shortest manner, to save your time; but it would be very easy to illustrate almost all these by hypothetical cases.

Sir F. THESIGER.—But I want to have on oath, your opinion now; that being your opinion, will you allow me to ask you this—"whether there are not a large number of patients who are properly confined in a lunatic asylum (I will take Hanwell, as an instance) who would be able to execute a deed, to count the money which they received, to work out simple rules of arithmetic and to conduct themselves in such a way as to lead even a professional person to believe that they were of sound mind as regards those acts?" A. No doubt there are many persons who are deranged who are still capable of certain exercises of intellect, those which you have mentioned. — Q. Have you found from your experience, that persons who have been in confinement in lunatic asylums, have been able to give a reasonable account of the grounds upon which they have been placed in confinement? A. Very often. — Q. But you have found those instances occurring in the course of your experience? A. Yes; certainly. — Q. Have you found that there are many persons who really conduct themselves with propriety when they are under the control and discipline of an asylum? A. There are many such. — Q. Did you converse with this lady upon the subject of her property at all? A. In the first interview which I had with her at York House, in September, 1846. — Q. Did you, upon these last occasions in December, and the other periods when you have seen her, converse with her about her property? A. I have not said a great deal about her property, but I have asked her, and reminded her that she was accused of living beyond her income, that that was one of the things alleged against her. — Q. Did you tell her she was accused of living beyond her income? A. That she was accused of spending more money than she could afford; I did not say accused, but that that was one of the allegations against her, because these allegations were the subject of every constant conversation on her part and mine.— Q Did you put it to her in that way? Do not suppose that I am imputing anything improper to you; I have too high a respect for your character and station, but I want to know (for it is important to know) how you put your questions. Did you say she was accused of living beyond her means? A. I will not say that I used the word "accused," but I certainly said that was one of the things alleged against her. — Q. What did she say about that? A. She did not say a great deal about that, certainly; she neither denied nor avowed it; she said, they said a great many things about her. — Q. I beg pardon for fastening upon an expression, but you said, she did not say a great deal about it herself, if some other person had? A. No. — Q. Did you ask her the particulars of her property, and what she had done with it? A. No, I did not. — Q. Did you ask her anything about her will? A. No.

Re-examined by Mr. James.—You have done me the honour to make me a present of this book; this appears to be a pamphlet which was elicited from you?

The Commissioner.—Are you going to put that pamphlet in?

Mr. James.—If a portion of the pamphlet be read to the jury, the fairer way is to let them have the whole.

Sir F. Thesiger.—I merely asked Dr. Conolly questions from his pamphlet in order to get his answer on oath.

Mr. James.—Very well, then I can do the same thing.—Is there anything in this pamphlet at all at variance with the opinion which you have expressed with reference to Mrs. Cumming's soundness of mind? A. I believe not one word.— Q. Is there anything in it which is in any way at variance with the result of your examination, and with what I need hardly describe as being your conscientious opinion as to the sanity of her mind? A. Nothing. There is nothing at variance with it. — Q. I believe that this pamphlet was elicited from you in consequence of the Lord Chief Baron laying it down that people ought not to be confined in a lunatic asylum unless they were dangerous to themselves and others? A. Yes. — Q. I will read the paragraph. "In the report of a recent trial," &c. (reads the passage.) I believe that your pamphlet was written to contest the proposition that persons ought not to be put in lunatic asylums unless they were dangerous to themselves and others? A. Yes; that was the origin of the pamphlet. — Q. I will assume a lunatic asylum a palace, and that every person in it is waited upon by six footmen—is it the comfort of a lunatic asylum which ought to influence a medical man in giving his opinion on the sanity or insanity of the patient? A. No. — Q. You state here that well regulated asylums are very proper receptacles for many persons of unsound mind? A. No doubt of it. — Q. Would you put, or advise the putting, any person of sound mind, but of feeble memory, into the very best regulated asylum? A. No. — Q. Or have you in this pamphlet suggested such a thing? A. No.

Sir F. Thesiger.—Do not let it be supposed that I have attributed any such intention to Dr. Conolly.

Mr. James.—You have been asked, if in an asylum there are many people who might execute a deed; there are many persons who are sane on some points, but with clear delusions upon others? A. Many. — Q. And are their actions sane, or their actions those of sane people, unless the existing delusion in some way or other interferes? A. There are many persons precisely of that description. — Q. But in those cases do you find the existing delusion haunting the mind upon some point? A. In a good number of cases the delusion haunts the mind; but I need not say there are many cases of insanity in which there is no delusion. — Q. Do you give a decided opinion as to the sanity of this lady? A. A very decided opinion. I always had the same opinion from the first interview; and every interview I have had with her has confirmed that opinion. — Q. You have been asked if you examined her and put questions as to her property; did you hear all the questions which the Commissioner and the Jury thought proper to put to her with reference to her property? A. I heard those questions. — Q. I presume there is a broad distinction between the partial failure of memory, and insanity? A. Quite so; it appears to me, that the state of Mrs. Cumming's mind at present in relation to that particular subject is this, that she could understand any single or plain proposition relating to any part of her property. — Q. I think you were saying on my putting the question, which was objected to by my friend, Sir F. Thesiger, that there is a broad distinction between feebleness of memory, or partial failure of memory, and insanity? A. Quite so. — Q. Have you found many persons in your experience, who, although they have a failure of memory as to their property, are of sound disposing mind and judgment when that property is put before them and brought to their attention? A. I am sure that that is the case with a great many persons, particularly at the time of life which Mrs. Cumming has attained. — Q. And is there not a broad distinction between a person having a delusion that he is possessed of a very large sum of money which he is not, and a failure of memory with reference to property of which they are really possessed? A. A very broad distinction.

The COMMISSIONER.—The way in which a person manages property may be a test of sanity or insanity? A. Yes, it may be a test. — Q. Is it not sometimes a test? A. There are such various shades—it may be a test of soundness and accuracy of judgment, without actual derangement of mind. — Q. Do you know, without referring precisely to what took place, how this lady has managed her property? A. No; I have no information upon that subject, except what I have heard in court. — Q. I am not going to ask you for a definition of delusion, but I will ask you this:—A. B. has a reason for believing that an attempt is made to murder him—that belief upon his mind is a delusion?

Mr. JAMES.—No; if he has reason for believing it, it cannot be a delusion.

The COMMISSIONER.—That is not a delusion—it is an existing fact? A. Yes. — Q. That is not insanity? A. No. — Q. Insanity is one thing, but a delusion is not insanity, it is only a test of it? A. Delusion often, I suppose, may be called a mere error of judgment, but a distinct delusion is a thing so very different that there can be no mistaking it; for instance, I would say that at Hanwell I have a number of persons, poor people, who imagine that they are going to be married to the Queen—that they are to be raised to the highest rank in the peerage to-morrow; and on the other hand, we have excellent persons who think they have been guilty of distinct crimes, which will be published in the *Times* newspaper to-morrow. — Q. Suppose a person has an idea that he is going to be married to the Queen, and that he has some ground for such a belief, that is not a delusion; but suppose the mind is altogether divested of that impression at a particular period, but that a year or two afterwards the mind reverts to the same impression without any fresh justifying cause, is that any test? A. I should say there was disease of the mind there. — Q. You must account for that I am afraid in one of two ways; either that the mind was never, in fact, divested of the original impression, or else that there must be something like a disease; am I right in that? A. Yes. — Q. If a gentleman has an impression that he is going to marry the Queen, from having received an anonymous letter, or something of that kind, that is intelligible, and it is no delusion; but it is no test of insanity; if, on the other hand, he is perfectly satisfied that that was altogether nonsense, and then the mind having got into that state without any cause arising whatever, the mind reverts back to the original impression, would you say that is a test of insanity? A. I should suspect it certainly.

A JURYMAN (to the witness).—Q. You have spoken of Mrs. Cumming's intensity of feeling; does that not generally exist in an unsound mind? A. Intensity of feeling, of course, according to the disposition of every individual, and there is no particular mark or line which you can draw; you must take a number of circumstances into consideration. — Q. It does not always exist? A. It does not exist in the same degree; but where it exists on certain facts we do not look on that as insanity, but as an excess of feeling, or perhaps an ill-governed feeling, according to circumstances. — Q. Do you think, from what you know of this lady, taking day after day liquors and wine, and things of that description, potent drinks that would cause at times a temporary derangement of intellect? A. I think it would cause a great variety of manner and mode of talking, and aggravate any eccentricity of disposition so as to account for some peculiarities. — Q. Do you think that at times she, from taking these potent drinks, is under temporary derangement of intellect? A. I have never seen her so. — Q. Nothing like delirium tremens? A. I have never seen her in any condition approaching to that, and I have never seen her in any state in which I have thought her under the influence of wine or spirits, never.

*Forbes Winslow, Esq. M. D.*, recalled, examined by Mr. James.—Q. There was a question I omitted to ask you last evening; there has been a suggestion, or an insinuation, made about this lady being tutored. You have heard the number of examinations from one side and the other, to which this old lady has been subjected? A. Yes, I have. — Q. And you have heard, perhaps, of the medical men going with the knowledge of what are alleged to be her delusions, and putting them distinctly to her, and hearing her answers and conversations on the subject? A. Yes. — Q. I will use the expression which has been insinuated, could she be tutored to conceal her delusions on these examinations? A. I do not think she could. — Q. For a period? A. For a short period. I do not think in Mrs. Cumming's case, she could. In cases of diseased mind, if you refer to the delusion, or touch the chord that is diseased, or out of tune, the delusion becomes generally immediately obvious.

Sir F. Thesiger.—That is what you asked Dr. Winslow yesterday.

Mr. James.—I was told, and was surprised to hear, that there had been some insinuations made, that this lady had been tutored to undergo these examinations.

Sir F. Thesiger.—No. The question was put to Sir Alexander Morison many days ago, whether she was not a person who was capable of being tutored to undergo the examination. I do not know why my friend should again call Dr. Winslow for this purpose.

Mr. James.—I have a right to recall the witness.

Sir F. Thesiger.—I beg pardon, it is not a matter of right; whenever it is done it is always with the permission of the Judge, who allows it, or not, as he thinks right.

A Juryman.—I do not put it that Mrs. Cumming had been tutored. I asked, whether a person might be tutored.

Mr. James.—It was not your question, sir, but the evidence given by Dr. Diamond and Dr. Davey, led to an insinuation, that she had been tutored in some way. — Q. Now I ask you, notwithstanding what a clairvoyant doctor may have said upon the matter, in your opinion could she be tutored to conceal her answers under any tutorage? A. It is quite impossible; in Mrs. Cumming's case it would be impossible; there are some cases where the lunacy on careful examination is not very apparent, but if the party examining has a key to the aberration, and touches upon the delusion, that must eventually become apparent.

The Commissioner.—Q. Supposing a man has reason to believe, from an anonymous letter, or anything of that kind, that he is going to be married to the Queen, that is not a delusion, that is not a test of insanity? A. Not taken by itself. — Q. Supposing a mind entirely gets rid of that belief for a considerable period, and the mind afterwards, without any new or fresh facts arising, reverts back to the same belief; in that case, should you say that mind is perfectly sane? A. I should not say that it would be an infallible test of insanity; it might excite a reasonable suspicion as to the condition of a person's mind. — Q. It raises some suspicion in our mind, nothing beyond that? A. No; it would excite suspicion. — Q. But you would not be satisfied, in the generality of cases, with one delusion, or any one fact, probably? A. If a delusion existed in the right acceptation of the term, one delusion, and it was clearly the result of a diseased condition of mind, I should have no hesitation whatever in saying that the mind entertaining it was unsound. — Q. You mean that one delusion of a strong character would be sufficient evidence to lead you to the conclusion that that man was of unsound mind? A. I do not confine myself to the mere *strength* of the delusion. Supposing, for instance, a person imagined his legs to be made of glass, and under that diseased impression or delusion, is careful how he moves his legs, from a fear that he might break the brittle article; that would be a delusion, irrespective altogether of its strength, degree, or influence upon the conduct of the person. — Q. It would be to a degree a symptom of an unsound and diseased mind? A. It is not a question of *degree* at all; if the delusion exists, it is a symptom of insanity. — Q. The delusion is only evidence o the disease of the mind? A. It is a symptom of a diseased mind. — Q. It is evidence, the same as if a man had no intellectual faculties ; it is not the disease, but it shows the existence of the disease? A. It is an indication of a certain state of mind to which the term insanity is applied. — Q. You know the case of a man who fancied that one leg was his own and the other Madame Vestris's, and saying, when asked to walk, that he could not do so, for that reason ; would that lead you to the conclusion that he was of unsound mind? A. I should have no doubt that he was of unsound mind if he entertained so absurd a notion.

Mr. James.—That, sir, is the case on the part of Mrs. Cumming.

